# Unravelling the role of transient redox partner complexes in P450 electron transfer mechanics

**DOI:** 10.1038/s41598-022-20671-0

**Published:** 2022-09-28

**Authors:** Tatiana Y. Hargrove, David C. Lamb, Jarrod A. Smith, Zdzislaw Wawrzak, Steven L. Kelly, Galina I. Lepesheva

**Affiliations:** 1 Department of Biochemistry, Vanderbilt University School of Medicine, Nashville, TN 37232 USA; 2 Faculty of Medicine, Health and Life Science, Swansea University, Swansea, SA2 8PP UK; 3 Synchrotron Research Center, Life Science Collaborative Access Team, Northwestern University, Argonne, IL 60439 USA; 4 Center for Structural Biology, Vanderbilt University, Nashville, TN 37232 USA

**Keywords:** Biochemistry, Computational biology and bioinformatics, Evolution, Molecular biology, Structural biology

## Abstract

The molecular evolution of cytochromes P450 and associated redox-driven oxidative catalysis remains a mystery in biology. It is widely believed that sterol 14α-demethylase (CYP51), an essential enzyme of sterol biosynthesis, is the ancestor of the whole P450 superfamily given its conservation across species in different biological kingdoms. Herein we have utilized X-ray crystallography, molecular dynamics simulations, phylogenetics and electron transfer measurements to interrogate the nature of P450-redox partner binding using the naturally occurring fusion protein, CYP51-ferredoxin found in the sterol-producing bacterium *Methylococcus capsulatus*. Our data advocates that the electron transfer mechanics in the *M. capsulatus* CYP51-ferredoxin fusion protein involves an ensemble of ferredoxin molecules in various orientations and the interactions are transient. Close proximity of ferredoxin, however, is required to complete the substrate-induced large-scale structural switch in the P450 domain that enables proton-coupled electron transfer and subsequent oxygen scission and catalysis. These results have fundamental implications regarding the early evolution of electron transfer proteins and for the redox reactions in the early steps of sterol biosynthesis. They also shed new light on redox protein mechanics and the subsequent diversification of the P450 electron transfer machinery in nature.

## Introduction

**Figure 1:**
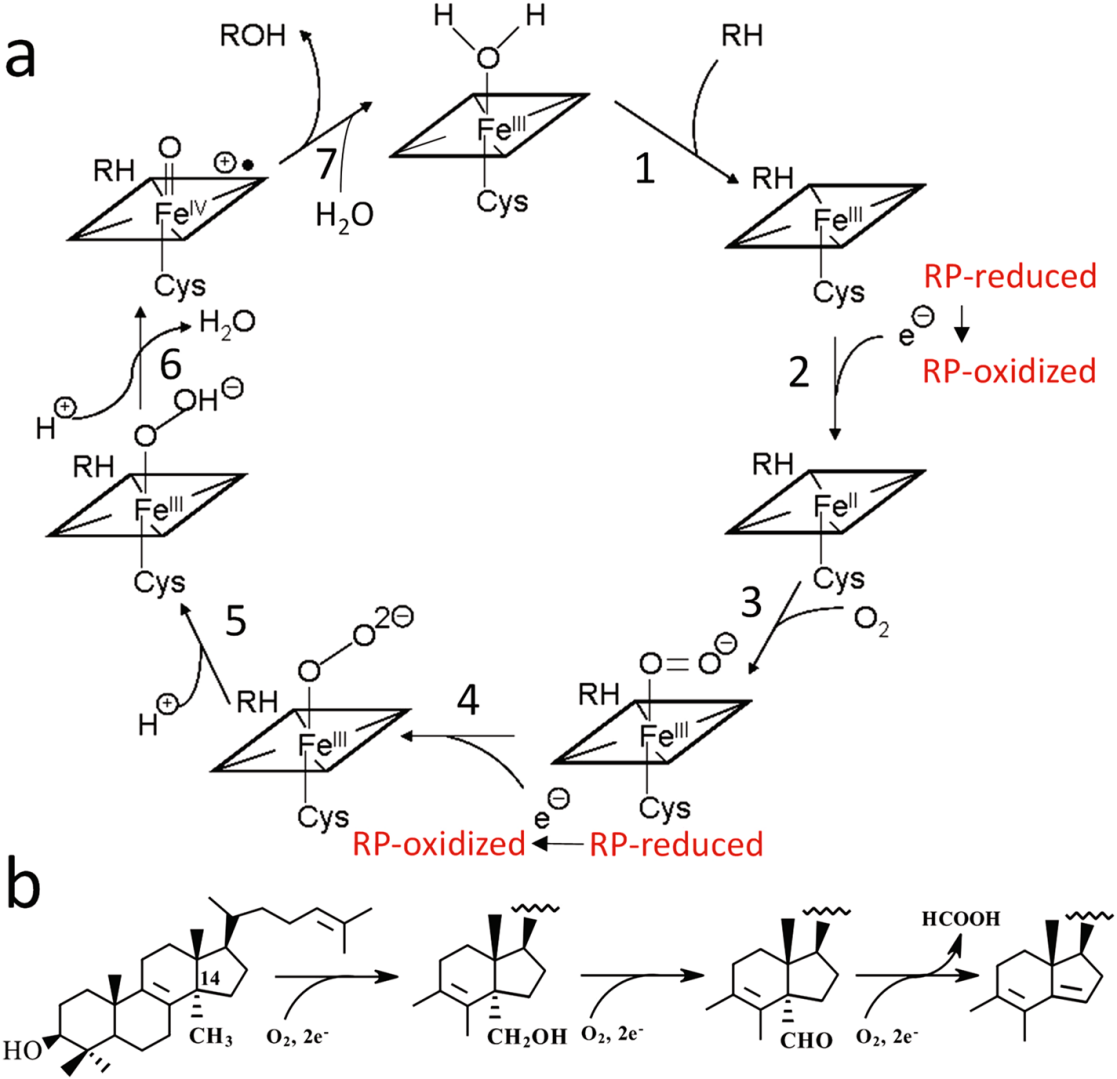
(**a**) Cytochrome P450 catalytic cycle. The cycle begins with the binding of a substrate, which increases the redox potential of the ferric heme iron (1). The iron (FeIII) accepts the first electron from the redox partner protein [RP], and the redox complex dissociates (2). The ferrous iron (FeII) binds molecular oxygen (3). The dioxygen complex accepts the second electron from the RP producing the ferric-peroxo anion and the redox complex dissociates (4). The ferric-peroxo anion is then protonated to form the ferric-hydroperoxo state (5). The second protonation of the distal oxygen atom causes the O–O bond scission, release of a water molecule, and generation of a highly reactive electrophilic ferryl (FeIV)-oxo cation radical known as Compound I (6), leading to insertion of the second oxygen atom into the substrate (7). (**b**) CYP51 reaction includes three P450 cycles and thus requires six sequential electron transfer events.

Cytochrome P450 (CYP, P450) enzymes constitute one of the largest gene superfamilies found in nature^1^. P450s occur in the *Bacteria*, *Archaea*, and the *Eukarya*; they have also been discovered in some viruses^2^. P450s have extremely diverse functions, playing essential roles in the metabolism of endogenous and exogenous molecules—ranging from the biosynthesis and degradation of sterols, vitamins, and various secondary metabolites to the detoxification and activations of drugs and pollutants^3,4^. At the molecular level, P450s are b-type heme-thiolate proteins that generally catalyze the molecular scission of atmospheric dioxygen, inserting one oxygen atom into the substrate whilst the second oxygen atom is reduced to a single water molecule (Fig. 1A).

During their catalytic cycle P450s require two electrons that are derived from the cofactors NADPH and/or NADH. The electrons are transferred, sequentially, to the P450 heme iron by ancillary (redox) partner proteins^5,6^. In *Bacteria* and *Archaea,* P450s examined to-date are soluble enzymes with the most frequent electron transfer (ET) chain being NADPH → FAD → [2Fe-2S] → heme Fe (FAD-containing ferredoxin reductase → the iron-sulfur cluster-bearing ferredoxin → P450, (class I)). Eukaryotes have two major P450 ET chains, depending on cellular localization. In the mitochondria, which presumably evolved from ancient bacteria^7^, the ET chain is essentially the same (NADPH → FAD → [2Fe-2S] → heme Fe), except that ferredoxin reductase and P450 are membrane bound while ferredoxin acts as a soluble shuttle. In the endoplasmic reticulum (ER), P450 redox mechanics is associated with the NAD(P)H-linked membrane-bound diflavin-containing reductase (CPR), ET proceeding via NADPH → FAD → FMN → heme Fe (class II). The advent of genome sequencing and the uncovering of the diversity of P450 enzymes from organisms across biological kingdoms established the biodiversity of P450-redox systems and highlighted many unusual forms. The first deviation from the class I/II standard described above was discovered by Narhi & Fulco who isolated a naturally occurring P450 fusion protein (CYP102A1; P450BM3) from the bacterium *Bacillus megaterium* consisting of a N-terminal P450 heme domain linked to a eukaryotic-like CPR containing FMN and FAD domains^8^. CYP102A1 is located in the cytosol and has enzymatic activity towards fatty acid hydroxylation with the highest turnover rate reported for any P450 monooxygenase (17,000 min^−1^). This is probably reflected in the rapid transfer of electrons between the fused domains compared with the separate components of the vast majority of P450 and redox partner proteins^9^. Consequently, it was thought that all P450-redox fusion proteins have evolved to maximize ET and catalytic efficiency of the P450 enzyme domain.

Sterol biosynthesis is generally associated with the eukaryotic kingdom of life, where the sterol end-product molecular structure differs, including cholesterol in animals, ergosterol in yeasts and fungi, and phytosterols in plants (for review^10,11^). However, the evolution of sterol biosynthesis is an active subject of investigation and vigorous debate. Though very few bacteria have been shown conclusively to synthesize sterol de novo, with the rapid advances in genomic and bioinformatic analysis the numbers of bacteria known to possess sterol biosynthetic genes are continually increasing^12^. The first bacterium proven to biosynthesize sterols was *Methylococcus capsulatus,* an obligatory methanotrophic gram-negative, non-motile coccoid bacterium^13^. *M. capsulatus* encodes a truncated sterol biosynthetic pathway which generates the sterols 4α-methyl-5α-cholest-8(14)-en-3β-ol, 4,4-dimethyl-5α-cholest-8(14)-en-3β-ol, 4α-methyl-5α-cholest-8(14)24-dien-3β-ol, and 4,4-dimethyl-5α-cholest-8(14),24-dien-3β-ol^14^. Presumably, the presence of sterols within this organism proffers a selective advantage to allow this bacterium to survive in the harsh environmental conditions that it inhabits.

Sterol 14α-demethylase (CYP51) is the cytochrome P450 that removes the 14α-methyl group from the sterol core (Fig. 1B). The *Cyp51* gene family is widely regarded as the evolutionary ancestor of all currently existing P450s, because its members are widely spread throughout all biological kingdoms, including > 1000 bacterial species from nine different phyla^15^. The *M. capsulatus* CYP51 enzyme was the first example of a P450 fusion protein that consists of a cytochrome P450 (CYP51) domain being linked at the C-terminus to a ferredoxin (fx) domain in a single polypeptide chain^16^. Recently, we identified large numbers of bacterial CYP51-fx fusions of similar structural organisation^15^, revealing that P450-redox partner fusions are widespread in prokaryotes.

Herein, we explore the evolutionary advantages of the bacterial CYP51-redox partner fusion in providing selective adaptation to their environment(s) as well as its possible ancestral role in diversification of the P450 electron transfer machinery. We have found that the ferredoxin domain in the *M. capsulatus* CYP51-fx fusion remains flexible even after binding of the substrate, with several poses of ferredoxin on the P450 surface having comparable interface energies and ET-efficient distances between the donor and acceptor atoms. This indicates that functional ET complexes in *M. capsulatus* CYP51-fx are transient. Our results imply that the fusion protein has evolved to allow the P450 domain to have access to its soluble redox partner in the protein-rich *M. capsulatus* membrane environment^17,18^ through fusion because there are no differences in ET efficiency in either the soluble or fusion protein states. Consequently, our results have implications regarding the early evolution of P450 ET through protein localization rather than catalytic efficiency and regarding mechanics and diversification of the P450 electron transfer machinery as a whole.

## Results

Previously, we crystallized the *M. capsulatus* CYP51-fx fusion in the substrate-free form^15^, and the electron density map indicated that ferredoxin was not in complex with the P450 domain but remained flexible. Our efforts in crystallizing the fusion protein with the physiological substrate, lanosterol, bound in the active site of the P450 domain were then undertaken in order to understand if the substrate binding in the P450 domain is accompanied by large structural rearrangements and, if so, whether these rearrangements stabilize the fused ferredoxin domain in a single (or at least in one major) conformation on the P450 surface to facilitate ET or whether the fusion evolved as a process to allow P450 access to redox protein without impacting ET.

### Substrate-induced conformational change in the CYP51 structure is conserved throughout phylogeny

**Figure 2:**
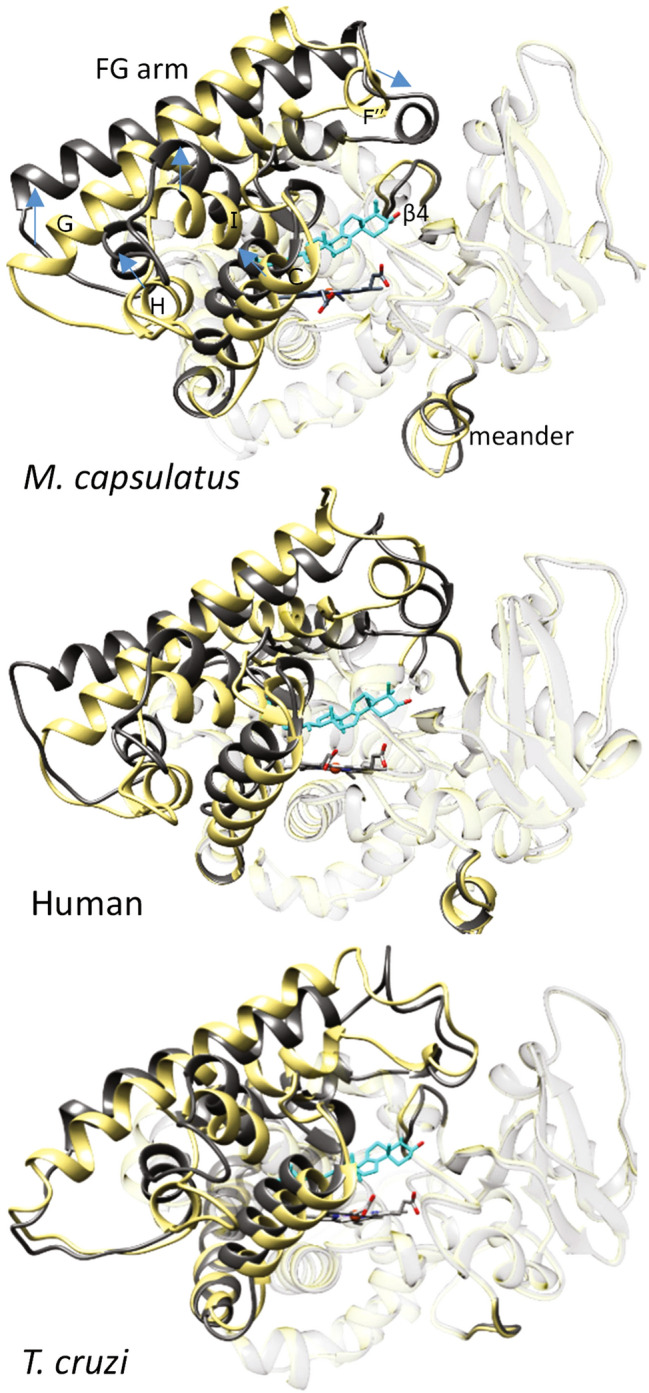
Substrate-induced conformational switch is similar in bacterial and eukaryotic CYP51s. Overlaid structures of substrate-bound (grey) and substrate-free (khaki) CYP51 orthologs. *M. capsulatus* (7SNM and 6MI0, respectively, rmsd of Cα of 2.44 Å), human (6UEZ and 4UHI, rmsd of Cα of 1.98 Å), and *T. cruzi* (6FMO, molecules A and D, rmsd of Cα of 1.83 Å). The orientation is about the same (upper P450 view). The directions of the changes are outlined with blue arrows on the *M. capsulatus* CYP51 structure. The carbon atoms of lanosterol (*M. capsulatus* and human) and obtusifoliol (I105F *T. cruzi*) are colored in cyan. The ribbons of the parts of the molecules that are not involved in the conserved conformational change^20,21^ (7SNM: rmsd of Cα of 0.77) are transparent. For comparison, the ligand-free and detergent-bound *M. capsulatus* CYP51 structures (6MI0 vs. 6MCW) have RMSD of Cα of 0.37 Å.

The lanosterol-bound *M. capsulatus* CYP51-fx fusion protein was crystallized in the P1211 space group, and the structure was resolved to 2.5 Å resolution. The asymmetric unit consisted of four molecules of P450, the active site of each containing one molecule of lanosterol (Supplementary Fig. S1, Table S1). As anticipated, binding of the substrate causes a large-scale conformational change in the P450 domain of *M. capsulatus* CYP51-fx fusion, which is very similar to that previously observed in the structures of two substrate-bound eukaryotic CYP51 orthologs, from *Trypanosoma cruzi* (6FMO)^19^ and human (6UEZ)^20^. Superimposition of the three structures and their sequence alignment can be found in Fig. S2, rmsd of Cα between the bacterial (7SNM) and eukaryotic orthologs being 1.53 Å (6FM0) and 1.56 Å (6UEZ). The rearrangements occur in several quite remote structural segments and substantially reshape the surface of the entire P450 molecule (Fig. 2). On the distal P450 face, the entrance into the substrate access channel, formed by helices A’, F’’ and the β4 hairpin, closes. This occurs due to the 7–10 Å movement and ~ 30° rotation of the FG arm that brings helix F’’ nearer to helix A’ and a 4 Å movement of the tip of the β4 hairpin. On the proximal (closest to the catalytic heme iron) P450 face, which is generally known to be involved in the interaction with the redox partner proteins, helix C moves ~ 6 Å inward, crossing the heme plane and approaching the lanosterol side chain. This moves helix H and the C-terminal portion of helix G ~ 8 Å along the same vector and relocates the N-terminal portion of helix I and the B’C loop, ~ 6 Å and ~ 3 Å upwards, respectively. Some rearrangements are also seen in the *M. capsulatus* (though not human or *T. cruzi*) meander region.

### Substrate binding mode and unique features discerned from the M. capsulatus CYP51-fx structure

**Figure 3:**
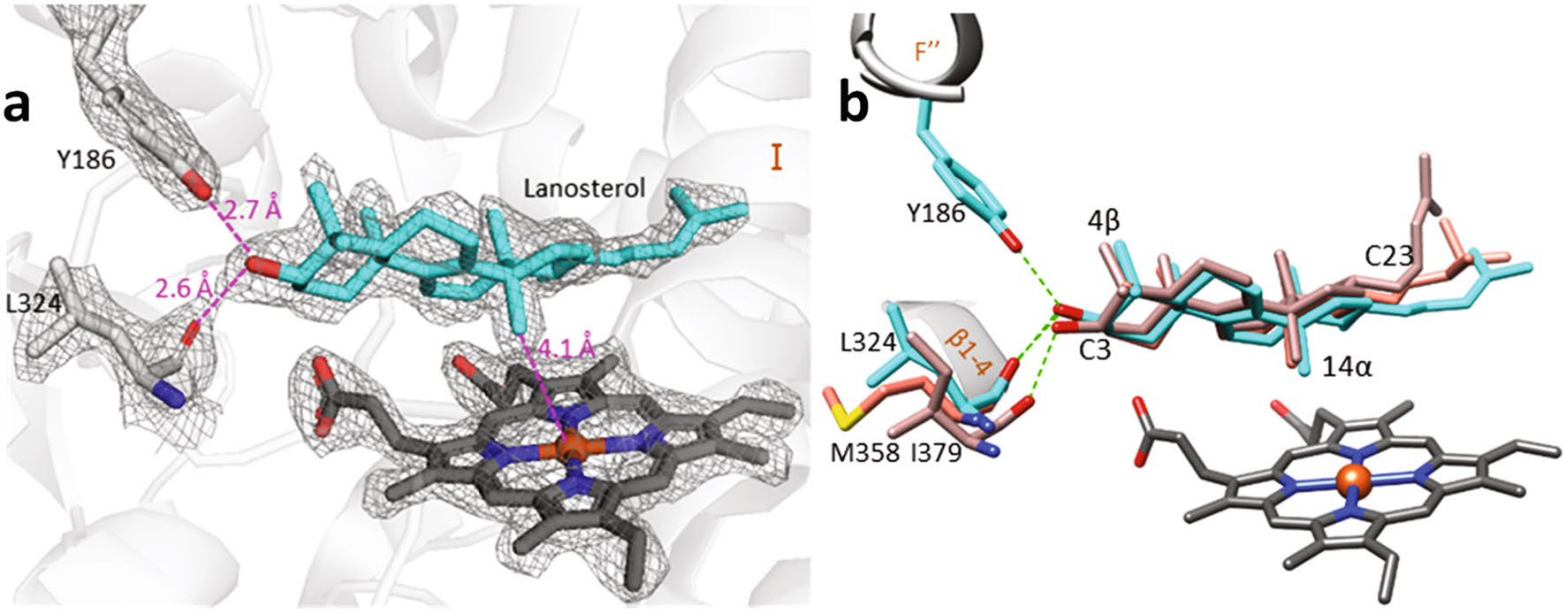
Binding mode of the CYP51 substrate is conserved across phylogeny. (**a**) The 2Fo-Fc electron density map (at 2σ) for lanosterol, the heme, and the H-bond forming residues, Y186 and L324, in the structure of *M. capsulatus* CYP51. (**b**) Sterol substrates bound in the active center of bacterial (*M. capsulatus*—cyan, and eukaryotic CYP51s (human—rosy-brown, and *T. cruzi*—salmon). The residues preceding the β1-4 strand (L324, I379, and M358) whose main chain carbonyl oxygen forms the H-bond with the sterol hydroxyl are colored correspondingly. The distances and the H-bonds are depicted as magenta and green dashes, respectively.

Inside the protein globule, the binding mode of the sterol substrates is also very similar (Fig. 3a,b), except that in the human CYP51 structure the lanosterol side chain is bent ~ 70° at C23 position. This conformation is likely due to the smaller volume of the human CYP51 active site. In all the three structures the sterol 14α-methyl group lies within 4.1–4.2 Å from the heme iron, i.e., they are clearly captured in the catalytically competent orientation. The 4β-methyl group is pointing upwards (toward helix B’). The C3-OH forms a hydrogen bond with the main chain carbonyl oxygen of the residue that precedes the β1-4 strand (L324 in *M. capsulatus*, I379 in human, and M358 in *T. cruzi* CYP51).

**Figure 4:**
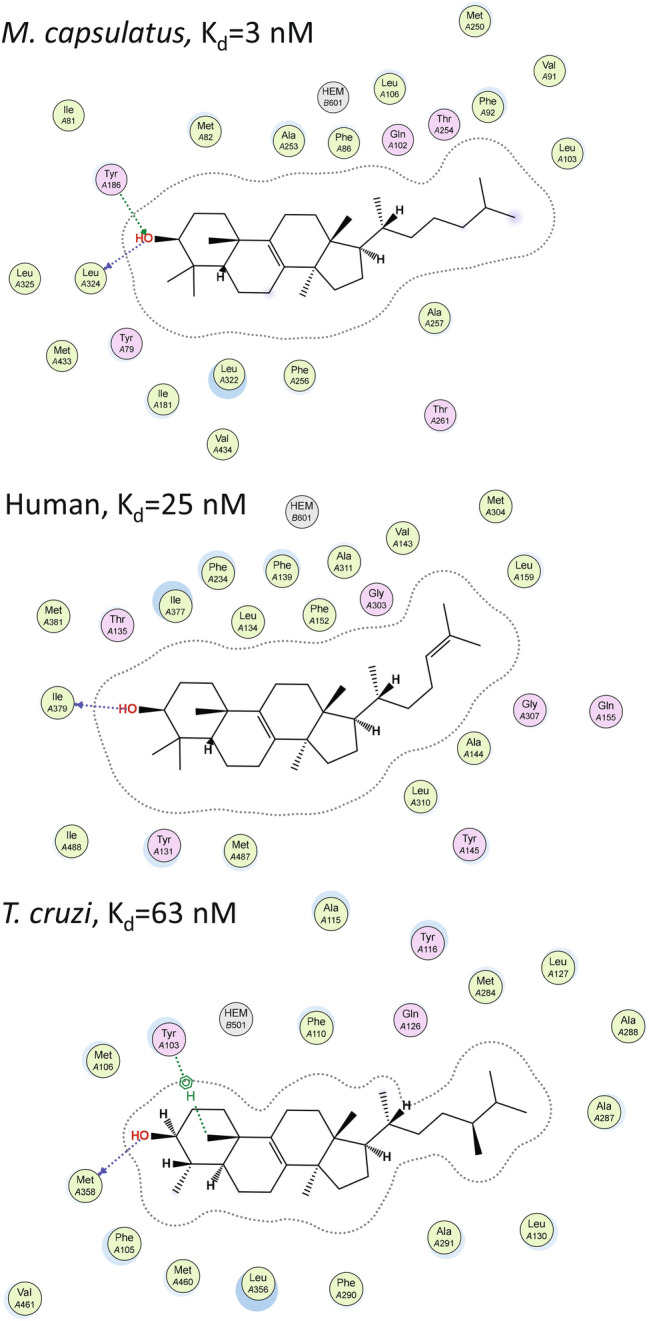
Interactions between the CYP51 proteins and their substrates. Calculations were made in MOE using molecule A. Polar interactions are in plum; hydrophobic interactions are in green, and the arene–H interaction between Y103 and C19 atom of obtusiofoliol is indicated as a green dotted line.

Closer examination reveals unique features for the *M. capsulatus* CYP51 structure. Firstly, there is an additional H-bond between the sterol C3-OH and the side chain OH of Y186 (located in helix F’’) not seen in eukaryotic CYP51s. This bond strongly implies that *M. capsulatus* CYP51 binds sterol substrates with higher affinity than its corresponding eukaryotic orthologs. Indeed, we found that there are no H-bond donors/acceptors in the F’’ helix in eukaryotic CYP51s, where this position is always occupied by a nonpolar residue: tryptophan in animals, phenylalanine in fungi, and valine in plants, algae, and protozoa. Furthermore, computational calculations of the substrate binding affinities in the three CYP51 structures in MOE (see Materials and Methods) predict Gibbs free energies (*ΔG*) of − 9.78 kcal/mol for obtusifoliol in the I105F CYP51 mutant of *T. cruzi*, − 10.35 kcal/mol for lanosterol in human CYP51, and − 11.62 kcal/mol for lanosterol in *M. capsulatus* CYP51 (where − 2.2 kcal/mol are provided by the H-bond with Y186). These values correspond to the *K*_*d*_s of 63 nM, 25 nM, and 3 nM, respectively (Fig. 4). Interestingly, the tyrosine residue in this position is rather conserved in bacterial CYP51s (though some of them have a phenylalanine here instead). This additional interaction is likely to be a reason for the previously reported substrate tolerance of *M. capsulatus* CYP51^15^, which so far is the only example of a characterized sterol 14α-demethylase enzyme that displays > 95% substrate occupancy (expressed as the low- to high-spin state transition in the heme iron) upon titration with each of the natural CYP51 substrates, lanosterol, eburicol, and obtusifoliol (Supplementary Fig. S3).

A second unique feature of the *M. capsulatus* CYP51 structure is that binding of the substrate does not free the side chain of E178 (helix F) from a salt bridge, an event essential to activate proton delivery in the eukaryotic CYP51 orthologs^19,20^. Although the H-bond with the CYP51 family signature histidine (the residue preceding the conserved P450 threonine in the I helix, H260 in *M. capsulatus*) is lost, a new and stronger salt bridge (2.8 Å) is now formed between the E178 hydroxyl and the neighboring H259 (this residue is unique and highly conserved in bacterial CYP51s^15^) (Supplementary Fig. S4). This indicates that, in contrast with the eukaryotic CYP51s structures, the presence of the substrate alone does not complete switching the *M. capsulatus* CYP51 domain to an active conformation.

**Figure 5:**
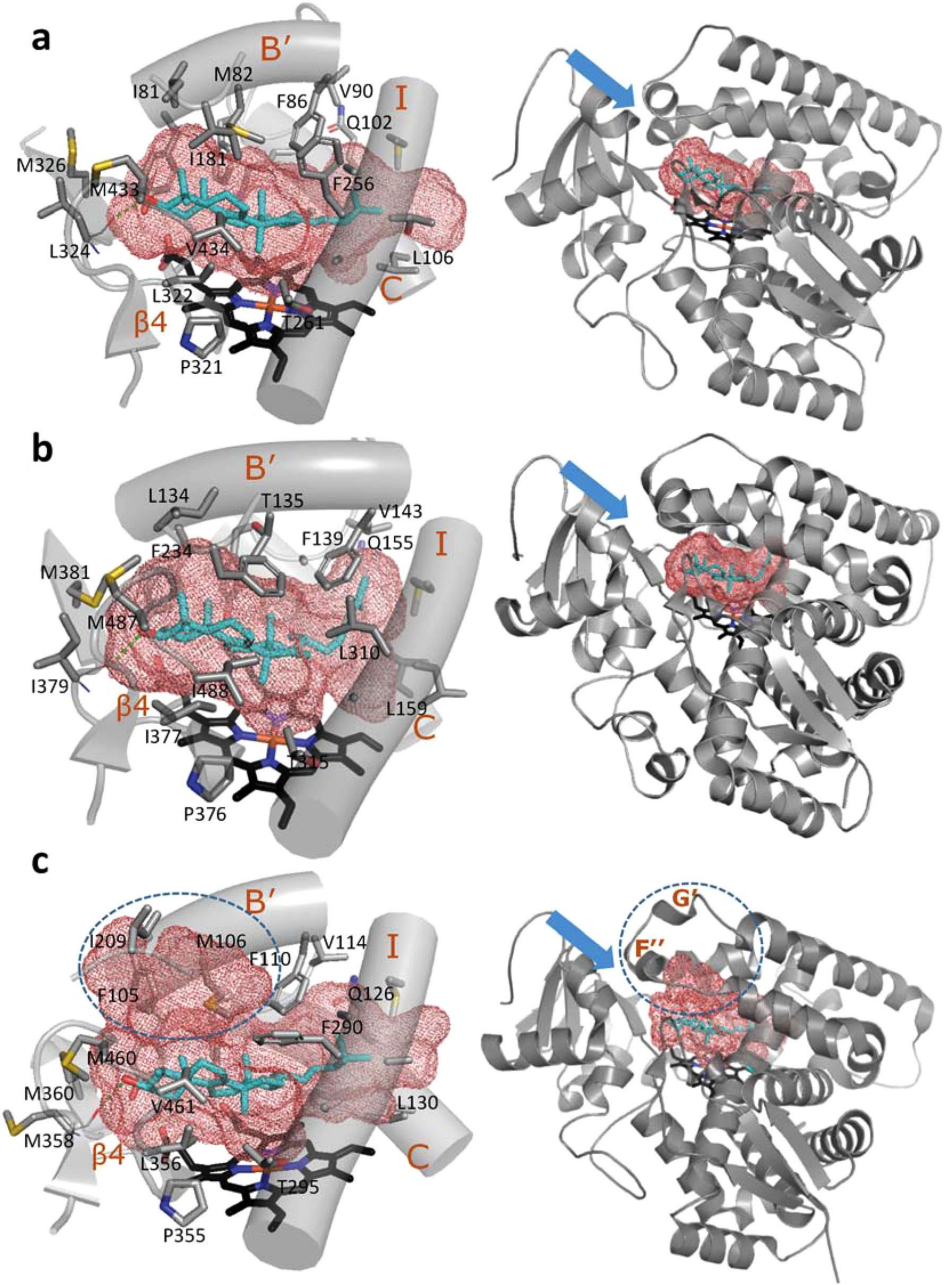
Substrate binding cavities in (**a**) *M. capsulatus*, (**b**) human, and (**c**) *T. cruzi* CYP51. Distal P450 view. Calculated in Voidoo, active site cavity volumes are 913 Å^3^, 865 Å^3^, and 1203 Å^3^, respectively. The substrate entrance^15,19–22^ is marked with a blue arrow, the subpocket in the active site of I105F *T. cruzi* CYP51 is circled.

Otherwise, despite the low amino acid sequence identities (~ 25%) between the three CYP51 proteins, structural elements of their active site cavity are strongly conserved (Fig. 5). The active site lies between helices B’, C, F’’, I, β strand 1–4, and β4 hairpin and is lined by 32, 36, and 29 amino acid residues in *M. capsulatus*, human and *T. cruzi* CYP51 structures, respectively (Supplementary Table S2). Most of them are hydrophobic in nature. Furthermore, 22, 18, and 21, of these residues, respectively (shown in Fig. 4), are located within van der Waals distances of the sterol substrate. Eleven of them (i.e., ≥ 50%) are invariant across the whole CYP51 family analyzed to-date, others are phyla-specific, and their side chain variations are reflected in the structural architecture of each enzyme active site. The larger CYP51 active site volume is seen in the *T. cruzi* structure (1200 Å^3^ *vs.* 865 Å^3^ in the human CYP51 and 913 Å^3^ in *M. capsulatus* CYP51 orthologs), and this observation is mainly due to the longer FG loop area in *Trypanosomatidae* CYP51s (these P450 sequences include an additional *Trypanosomatidae*-specific helix G’^21^). This region of the active site in *T. cruzi* CYP51 looks more like a “sub-cavity”, perhaps even reshaped as a result of the I105F mutation. Overall, the comparative structural analysis supports the notion^22^ that the conserved catalytic function of sterol 14α-demethylases is maintained across phylogeny via conservation of their substrate-binding cavity.

### Electron transfer from ferredoxin to P450 in the *M. capsulatus* CYP51-fx fusion occurs via multiple transient interactions

It was assumed that lanosterol bound into the *M. capsulatus* CYP51-fx crystal structure can serve as a molecular trigger to resolve the P450-ferredoxin complex due to the anticipated conformational rearrangements involving the proximal surface of the P450 molecule. Despite these readjustments, however, the electron density for the ferredoxin domain (or even the [2Fe-2S] cluster) in the structure of the fusion protein was still absent (Supplementary Fig. S5). Our attempts to construct at least a part of the ferredoxin molecule manually, including utilizing various docking poses as references, indicated that the P450-ferredoxin fusion protein complex is not strong enough to keep the electron donor partner interacting with P450 surface in a single orientation, at least during the crystallization process. This is in direct contrast to the single orientation observed in the other naturally occurring bacterial P450 fusion protein CYP102 (BM3)^23^, which could be because BM3 exist as a dimer in the solution and it was recently shown that the P450 domain of each polypeptide chain interacts with the FMN-binding domain of another chain^24^), while *M. capsulatus* CYP51-fx in the solution is monomeric (SEC chromatography). The fact that the interactions between the P450 and ferredoxin domains in the *M. capsulatus* CYP51-fx fusion protein are weak is also supported experimentally. The sterol 14α-demethylase activity of either the complete fusion protein or the engineered separate P450 domain is efficiently reconstituted with the *E. coli* flavodoxin/flavodoxin reductase system [the ET chain NADPH → FAD → FMN → heme] in both states^15^. Our findings strongly point to a hypothesis that the *M. capsulatus* CYP51-fx fusion did evolve as a mechanism to guarantee ET to drive the complex three-step (six electron transfers (Fig. 1)) reaction of this ancient P450 catalyst.

**Figure 6:**
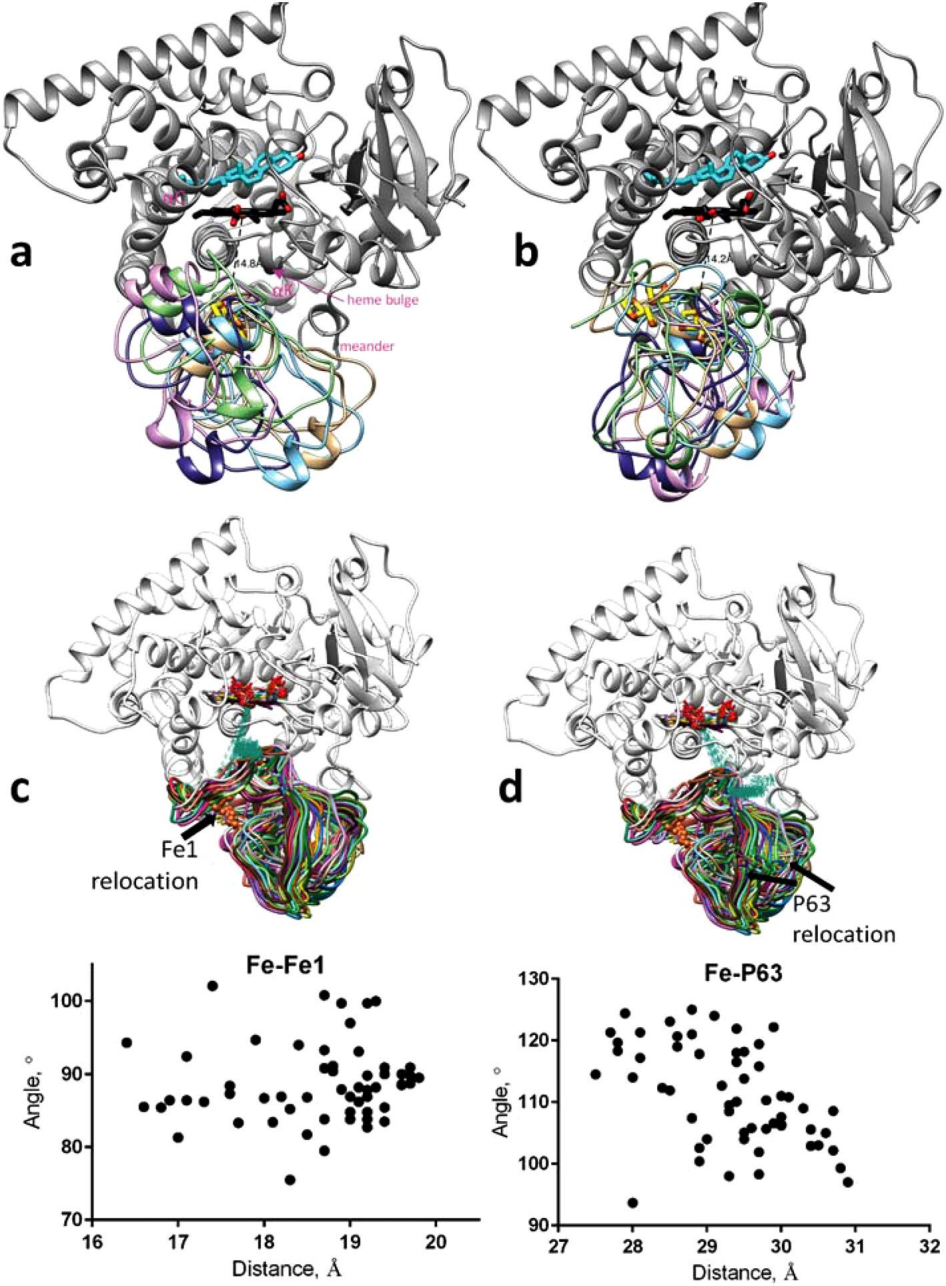
*M. capsulatus* CYP51-ferredoxin complex. Upper P450 view. (**a,b**) Five poses of the ferredoxin domain docked to the proximal surface of the P450 domain (grey), (**a**) docking in MOE, the heme iron to the Fe1 atom of the [2Fe-2S] cluster distance (|Fe–Fe|) range is 14.1–16.4 Å, (**b**) docking in Rosetta, |Fe–Fe| range is 13.3–16.7 Å. Lanosterol, heme, and [2Fe-2S] clusters are shown as stick models. (**c,d**) The MD snapshots of P450-superimposed complexes, showing changes in the distances/angles between (**c**) the heme iron/heme plane and Fe1 of ferredoxin and (**d**) the heme iron/heme plane and P63 Cγ atom of ferredoxin throughout the course of 600 ns simulations. The corresponding molecular dynamics trajectories can be seen as Supplementary Fig. S6.

Protein–protein docking analysis undertaken herein revealed several *M. capsulatus* CYP51 ferredoxin orientations that were compatible with efficient ET to the P450 domain. The highest score poses of the ferredoxin domain docked in MOE (the distance range between the heme iron and the iron atom (Fe1) closest to the surface in the [2Fe-2S] cluster of 14.1–16.4 Å) suggested that the polypeptide chain of ferredoxin is rotating, while the position of the [2Fe-2S] cluster does not change significantly (Fig. 6a). The highest score poses in Rosetta (the distance range of 13.3–16.7 Å) were even more variable, involving not only the polypeptide chain rotations but also “sliding” of the ferredoxin [2Fe-2S] cluster over the proximal P450 surface (Fig. 6b). This probably reflects the Rosetta docking algorithm which includes additional sampling of the protein conformational space in a search for the lowest binding interface energy. Calculations using the Marcus Eq. ^25^ (see Materials and Methods) estimated the ET rates to range from 1.7 × 10^2^ s^−1^ to 4.0 × 10^3^ s^−1^ and from 1.1 × 10^2^ s^−1^ to 1.2 × 10^4^ s^−1^ for the MOE and Rosetta poses, respectively (the latter corresponding to the distances of 14.1 Å and 13.3 Å). In both instances the ET is clearly sufficient to support *M. capsulatus* CYP51 catalysis.

**Figure 7:**
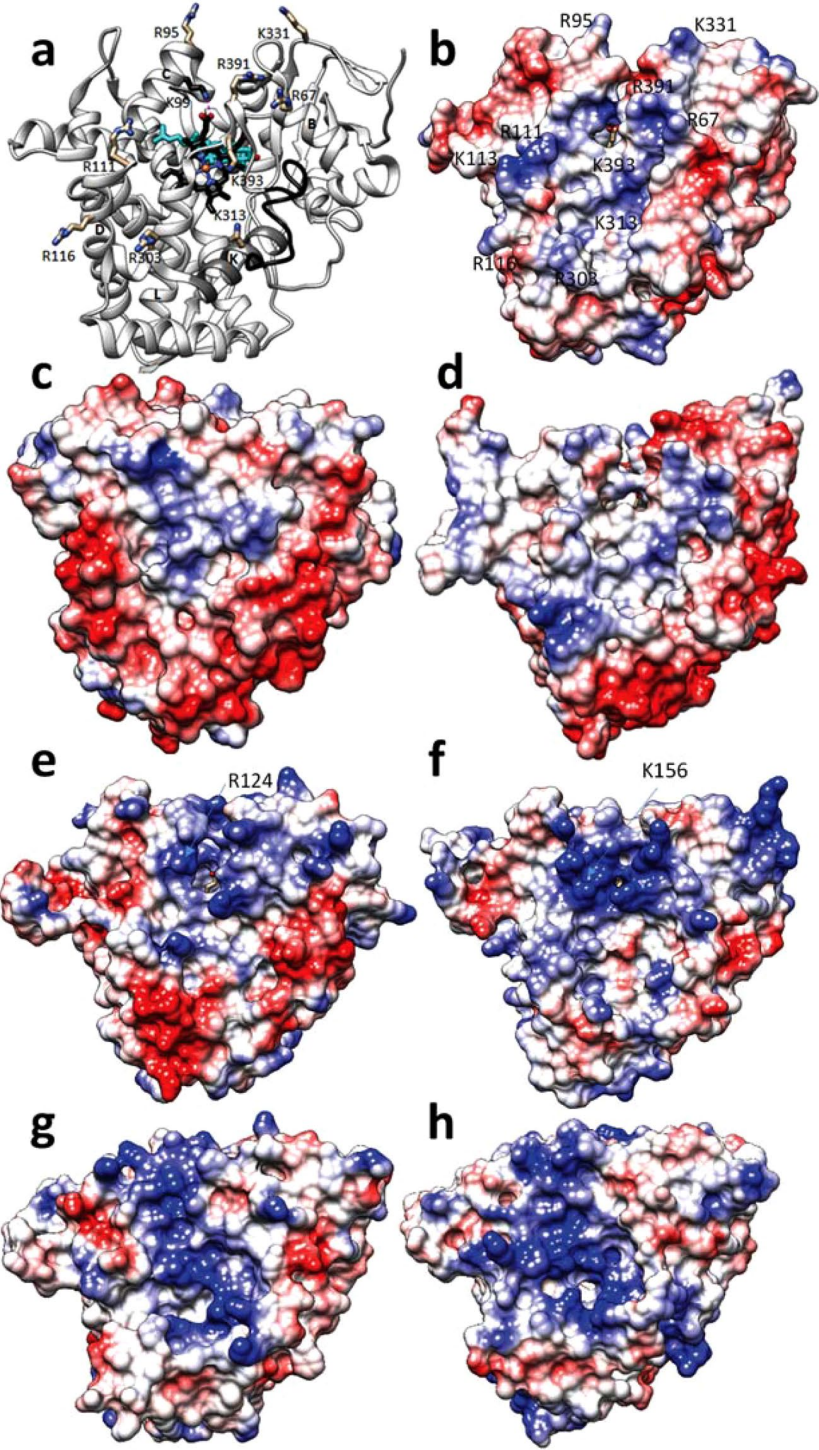
Proximal P450 surface. (**a**) Positively charged residues in the lanosterol-bound *M. capsulatus* CYP51 molecule. The ribbon of helix K (306–318) is colored in dim grey, the meander (370–385) is in black. The heme and lanosterol are shown as black and cyan stick models, respectively. K99 (colored in black) is not exposed to the surface, its side chain retains the H-bond with the heme ring D propionate. (**b–f**) Electrostatic potential mapped onto the surface of bacterial (**b–d**) versus eukaryotic microsomal (**e**,**f**) and mitochondrial P450s (**g**,**h**). (**b**) *M. capsulatus* CYP51 [7SNM], (**c**) *Tepidiphilus thermophiles* CYP116B46 (P450_TT_, 6LAA), (**d**) *M. tuberculosis* CYP51 [1E9X], (**e**) *T. cruzi* CYP51 [6FMO], (**f**) human CYP51 [6UEZ], (**g**) human CYP11A1 [P450scc, 3N9Y], (**h**) CYP11B2 [7M8I]. Red for positive and blue for negative charge, white for neutral. The view of the superimposed structures is the same as in (**a**).

We believe that the Rosetta predictions must be more reflective of the P450-fx interactions for two reasons: (1) there is no clear density for the bulky iron-sulfur cluster in the electron density map and (2) positively charged residues on the proximal surface of the *M. capsulatus* CYP51 domain are not localized around the heme but distributed quite randomly (Fig. 7a). Thus, the *M. capsulatus* P450 K helix (residues 305–318), the helix known to form the major portion of the ferredoxin binding interface in human mitochondrial P450s, CYP11A1 (P450scc)^26^ and CYP11B2^27^, contains only one surface exposed positively charged residue, K313. Moreover, in all CYP51 structures solved to-date (from *M. capsulatus* to human) helix K lies beneath the meander region, which makes its involvement in the protein–protein interactions unlikely. Two other surface-exposed positively charged residues in *M. capsulatus* CYP51, R95 and R111, are from helix C (residues 95–111). Helix C forms the major region of the ferredoxin binding interface with P450cam^28,29^. R116 is from helix D (113–131), R303 is from helix J’ (298–304), K331 is from the β1-4/β2-1 turn (330–332), R391 and K393 are from the heme bulge (389–393), and R67 is from helix B (59–68). Of interest here is K99, the residue that corresponds to the heme propionate-binding R124 in *T. cruzi* CYP51, K156 in human CYP51, and R112 in P450cam. Upon binding of the substrate all these three residues were shown to lose their contact with the heme, the side chain flipping up towards the proximal P450 surface and playing a role in the ET process^19,20,28^. In the lanosterol-bound *M. capsulatus* CYP51, however, K99 does not change its position but retains the H-bond with the heme propionate so that its positively charged guanidine group remains buried inside the protein globule.

The K99 residue also does not flip upon molecular dynamics (MD) simulations of the CYP51-ferredoxin complex. In 14% of the MD snapshots, however, it loses its H-bond with the heme. Interestingly, when ferredoxin is in ET efficient proximity to the P450 domain, the H259-E178 salt bridge opens in 45% of the MD snapshots (Supplementary Fig. S4). This resembles the conformational rearrangements induced by putidaredoxin in the crystal structure of bacterial (*Pseudomonas putida*) P450cam^28,30,31^.

Otherwise, the results of our MD calculations also support the idea of some flexibility in the ferredoxin orientation on the *M. capsulatus* CYP51 molecule, showing that both the distance between the heme iron and the proximal iron (Fe1) of the iron-sulfur cluster and the angle between the heme plane and Fe1 (Fig. 6c) as well as the distance between the heme iron and a randomly selected ferredoxin surface residue (P63, atom Cγ) and the angle between the heme plane and P63 (Fig. 6d) are changing readily and do not converge to one conformation after 600 ns of simulation time. Regardless of these transient interactions, the docking models and subsequent MD simulations are consistent with a possible electron transfer path in *M. capsulatus* CYP51-fx (Fe1 → Glu19 → Lys393 → Cys394 → Heme Fe (Supplementary Fig. S7)), as these residues are involved in high-scoring protein–protein interfaces (in the top 0.7% of all decoys) and remain in close proximity throughout our simulations.

## Discussion

The possibility that the ferredoxin domain is sliding and rotating over the proximal surface of *M. capsulatus* CYP51 domain is also supported by examination of the electrostatic potential of this P450 (Fig. 7b), whereby positive charge is both widely distributed across the whole proximal surface and rather moderate. Comparison of *M. capsulatus* CYP51-fx P450 domain with the structures of other P450s reveals that, in terms of overall electrostatic potential on the proximal surface, P450s display a very low extent of conservation. For example, the low positive charge on CYP116B46 (a self-sufficient bacterial enzyme where the P450 domain is fused both to ferredoxin and ferredoxin reductase in a sequential order) (Fig. 7c) somewhat justifies the long distance (~ 30 Å) between the ferredoxin [2Fe-2S] cluster and the P450 heme iron observed in the crystal structure^32^. Low positive charge is also seen on the proximal surface of CYP51 from *M. tuberculosis* (Fig. 7d). Though this P450 is expressed as a separate, non-ferredoxin fused, protein in this bacterium, a possibility exists that in an ancestral bacterium, now lost in time, there could have been a single ancestral gene encoding P450-ferredoxin as a single polypeptide chain. In the *M. tuberculosis* genome, and many other *Mycobacteria* sp., the CYP51 and ferredoxin genes are separated by only five nucleotide bases^33^. The connection between both genes could have been lost e.g., as a result of a stop codon inserting mutation, yet the surface charge remained low, because of the lack of evolutionary pressure due to loss of function in vivo. The questions at what evolutionary stage did the CYP51-ferredoxin fusion occur and whether eukaryotic CYP51s evolved from a fused bacterial ancestor remain open to debate. It is known, however, that prokaryotic adrenodoxin/putidaredoxin-like [2Fe-2S] water soluble ferredoxins, like native CYP51-ferredoxin fusions^15^, are mostly found in Proteobacteria^34^.

Eukaryotic CYP51s display a substantial increase in the positive electric charge on the proximal face, not just in comparison with the bacterial orthologs, but also across kingdoms from protozoa to human (Fig. 7e,f). Though topologically different, the K and R residues in both these CYP51 proteins cluster close to the center and above the P450 heme plane. This may reflect the fact that they are microsomal P450s that use CPR and not ferredoxin as a redox partner. The protein surface positive charge of human mitochondrial CYP11A1 (P450scc) and CYP11B2 (Fig. 7g,h), which represent another example of ferredoxin driven P450 monooxygenases, is even higher. A distinct patch of charged residues is crossing the proximal surface from the top (helix C), via the heme bulge and down to helix K and the meander.

Ultimately, we surmise that in the bacterial P450-ferredoxin fusion proteins, where the ferredoxin domain is attached to the C-terminus of the P450 domain, there is no evolutionary advantage for one preferred orientation as the ferredoxin domain can always deliver electrons to their attached P450 via random encounter complexes. In contrast, eukaryotic (both mitochondrial and microsomal) P450—redox partner interactions probably evolved to use stronger electrostatic contacts that stabilize one preferred binding conformation and/or binding site. On the other hand, it remains unclear whether the ferredoxin orientations captured in the crystal structures of the complexes are indeed optimal for ET in vivo. This is because we see in the structures that the thermal motions for ferredoxin atoms are high (indicating dynamic mobility) and because the artificial fusion P450-ferredoxin proteins that have been constructed for crystallization have either very low (> 200-fold decrease^26^) or no activity unless a large (40-fold) molar excess of additional (non- fused) ferredoxin is added to the system^27^. One must also consider the alternative possibility that other, though less populated, redox partner orientations are also involved in the ET process.

The nature of the interactions between a P450 and its electron donor protein(s), as well as the extent of evolutionary conservation of the recognition sites have been under discussion for decades^35–40^. By now it is assumed that combinations of predominantly electrostatic interactions (via basic residues on the P450 surface and acidic residues on the redox partner surface) and to a lesser degree hydrophobic and van der Waals contacts, are responsible^41^. The impact of each of these individual residues and the strength of the overall protein complex appears to vary from one P450 to another^37,42^. However, by definition, the complex where the components associate and dissociate temporarily in vivo cannot be too strong, since the interactions are broken and formed continuously. Thus, the question as to whether a redox partner adopts a single most efficient orientation for each individual P450 protein or whether the ET process occurs via random transient encounter complexes remains open for continued scientific debate and scrutiny. At least in the case of *M. capsulatus* CYP51-fx the results of our work suggest that the ET mechanics involves an ensemble of ferredoxin molecules in various orientations.

**Figure 8:**
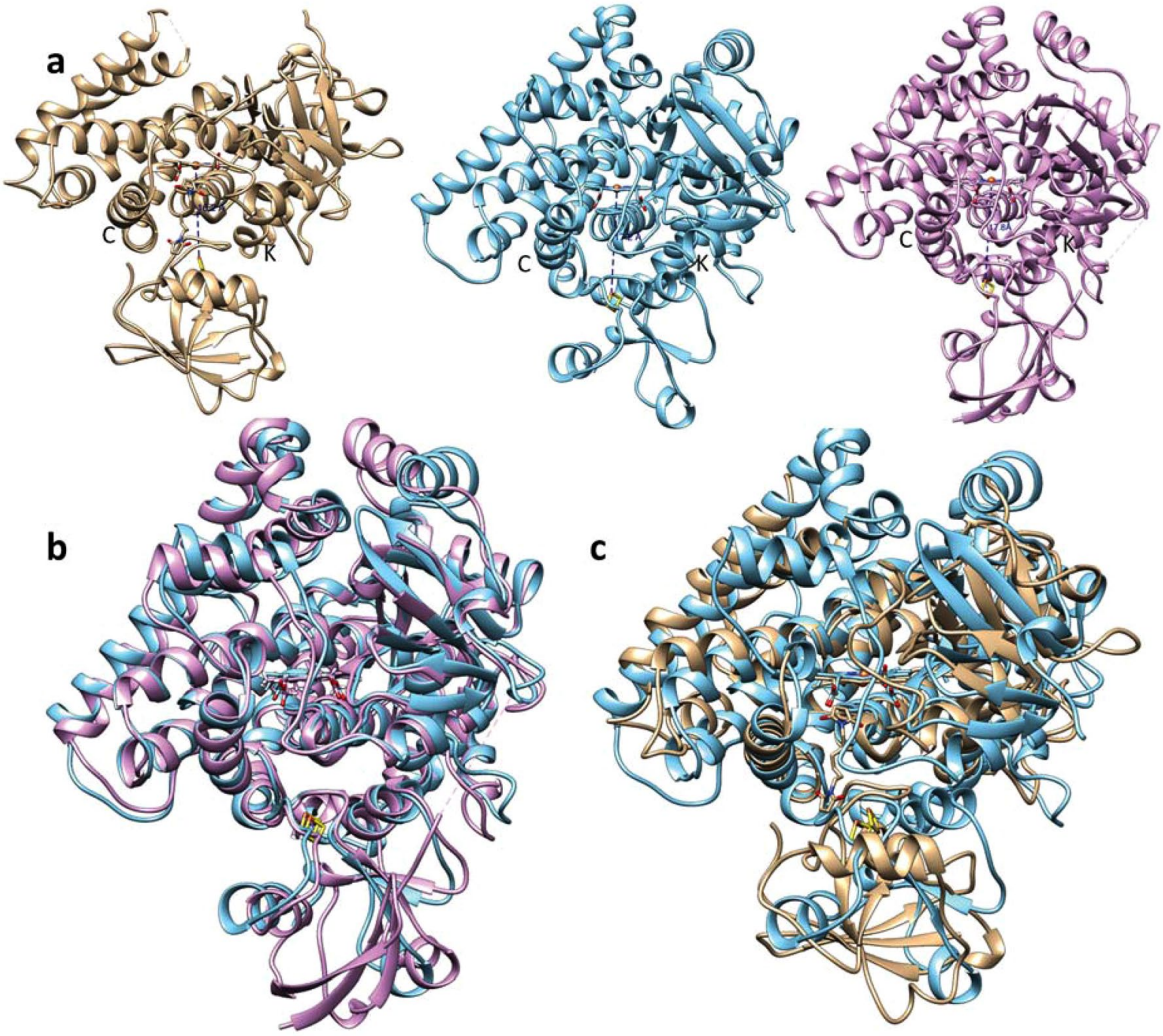
Crystal structures of P450-redox partner complexes. (**a**) Bacterial (*Pseudomonas putida*) P450cam with putidaredoxin [4JWS, tan] and human mitochondrial P450s with adrenodoxin: CYP11A1 [3N9Y, cyan] and CYP11B2 [7M8L, plum]. P450 orientation is the same. The distances between the Fe1 and the heme are marked. (**b,c**) Overlaid 3N9Y/7M8I (**b**) and 3N9Y/4JWS (**c**).

To date, the crystal structures of only four P450s linked with ferredoxin have been reported. Chronologically, the first structure was of human CYP11A1 (P450scc, 3N9Y) with the density of a fragment of bound ferredoxin molecule (the [2Fe-2S] cluster and residues 28–95, metal-to-metal distance (|Fe–Fe|) of 17.4 Å). Here the expression construct produced an artificial fusion protein comprising an N-terminal adrenodoxin domain linked to a C-terminal P450 domain^26^. The second structure was of bacterial *Pseudomonas putida* CYP101A (P450cam), covalently cross-linked (4JWS, |Fe–Fe| of 16.2 Å)^28^ and then noncovalent but modified using a series of P450/ferredoxin surface mutations (3W9C, |Fe–Fe| of 16.3 Å)^29^. The third was the structure of bacterial fusion P450116B46 from *Tepidiphilus thermophiles* (P450tt-ferredoxin-ferredoxin reductase) contains ferredoxin but it is bound not to the P450 but to the reductase (6LAA, |Fe–Fe| of 30 Å)^32^. The most recent structure is of another human mitochondrial P450, CYP11B2 but which is also as an artificial fusion adrenodoxin-P450 complex, with 104 ferredoxin residues and the strongest interactions in the binding interface (7M8I, |Fe–Fe| of 17.8 Å)^27^. Interestingly, in each of these protein structures the ferredoxin domains have different orientations relative to the P450 domains (Fig. 8a). Thus, in the CYP11B2 complex (Fig. 8b), the ferredoxin domain is shifted ~ 3 Å and rotated 10° in comparison with its position in the CYP11A1 complex and with different residue pairings^27^. In the P450cam, the ferredoxin domain is rotated 180° relative to its position in the mitochondrial complexes (Fig. 8c).

For the naturally occurring *M. capsulatus* CYP51-fx protein, it is possible that the evolutionary advantage of the fusion event is not an optimized ET rate, which is relatively slow in comparison to other naturally occurring P450-redox fusion proteins, but guaranteed electron transfer in a single polypeptide.

Thus, as is discussed above, *M. capsulatus* CYP51-fx binds its sterol substrate with stronger affinity than the characterized eukaryotic CYP51 orthologs—the additional Y186 H-bond [conserved in bacterial CYP51s], and the ferredoxin domain is fused, so that this synergistic combination delivers electrons efficiently to the neighboring P450 domain to ensure catalysis. Consequently, the P450-ferredoxin complex can exist in a transient nature, the ET is relatively slow but reliable. Later, faster catalysis became more preferable as the pathway evolved in different domains of Life, the sterol substrate diversified and binding weakened, the ferredoxin domain became "independent" and more redox partner proteins (including multiple ferredoxins) became available, the P450 proximal surface became more positively charged and so preferred ET partner orientation(s) became more diverse. All these evolutionary processes resulted in swifter P450 ET and faster catalytic rates.

## Materials and methods

### Protein purification and characterization

The protein was expressed in *E. coli* (HMS-174, Novagen), solubilized from the membrane fraction with 1% Triton X-100, and purified in two steps, including affinity chromatography (Ni^2+^-NTA agarose) followed by cation exchange chromatography (CM-Sepharose) as described^15^. The purity and molecular weight were assessed by SDS-PAG, which revealed a single band at the expected molecular weight of ~ 63 kDa (561 amino acid residues, including 451 residues of the P450 domain, 20 residues of the linker, 80 residues of the ferredoxin domain, and a 10 residues His tag at the C-terminus^16^). Catalytic activity was confirmed using *E. coli* flavodoxin/flavodoxin reductase system, the ability of the *M. capsulatus* ferredoxin domain to transfer electrons to the P450 domain was confirmed using spinach ferredoxin reductase, and the apparent sterol substrate binding affinities for the enzyme in the solution were calculated from the spectral titration curves and fit with a quadratic binding (Morrison) equation in GraphPad Prism (San Diego, CA).

### Protein crystallization

Since, based on our previous experience, upon crystallization the substrate can leave the CYP51 active site, e.g., being replaced by a detergent, which is required for crystallization to maintain the protein in its monomeric state^15^, prior to data collection crystals were dissolved in 20 mM potassium phosphate buffer, pH 7.6, containing 200 mM NaCl and 10% glycerol, and the high spin state of the heme iron was verified spectrophotometrically. The absorbance spectra were recorded using a NanoDrop spectro-photometer (Thermo Fisher Scientific). At the conditions described below, the Soret band maximum remained at 393 nm. Correctness of the crystallized protein molecular weight was verified by SDS-PAGE and ruled out proteolytic cleavage.

To prepare the substrate-bound sample, the protein was diluted to the concentration of 7 μM with 20 mM potassium phosphate buffer, pH 7.6, 10% glycerol, 0.1 mM EDTA, and 500 mM NaCl, then mixed gradually with 0.5 mM lanosterol solution in 45% (w/v) 2-hydroxypropyl-β-cyclodextrin (HPCD), molar ratio 1:2.5. The mixture was incubated 20 min at room temperature, centrifuged at 16 000 g to remove any precipitate, concentrated to 750 µM using Amicon Ultra centrifugal filters (Ultracel 50 K) (Millipore,Germany), and then diluted to 250 µM with 5 mM potassium phosphate buffer, pH 7.4, prior to addition of the detergent dodecyltrimethylammonium chloride (Hampton Research), final concentration 92 µM (2 cmc). The lanosterol-bound crystals were obtained by the sitting drop vapor diffusion technique. The crystallization drop consisted of 8 µl of the protein solution overlaid with 4 µL of reservoir solution (0.1 M Na-citrate tribasic dehydrate, pH 7.1, and 16% PEG 8,000) and equilibrated against the reservoir solution at 20 °C. Crystals appeared after a week, harvested, transferred into the mother liquor with 25% glycerol (v/v) and flash-cooled in liquid nitrogen.

### Structure determination, refinement and analysis

X-ray diffraction data were collected at 100 K on beamline 21-ID-D (1000 nm) of the Advanced Photon Source, Argonne National Laboratory, indexed and integrated with autoProc^43^, and scaled with Aimless^44^. The structure of the lanosterol-bound P450 domain was solved by molecular replacement using PhaserMR in the CCP4 program suite^45^ and the structure of the ligand-free P450 domain (PDB code 6mi0) as a search model. The refinement and model building were performed with Refmac5 (CCP4) and Coot^46^, respectively, and gave the structure of 443 P450 residues. Lanosterol and the heme were fitted into the difference map. Several iterations of model building in Coot and refinement in Refmac finalized the structure, with Rfactor and Rfree of 0.20 and 0.24, respectively. RMSD values of bond lengths and angles from the ideal values were 0.002 Å and 1.3°, respectively. The data collection and refinement statistics are shown in Supplementary Table S1. Structural comparisons were accomplished and RMSDs calculated in LSQkab (CCP4) using secondary structure matching algorithm. The active site depiction and volume calculations were performed in Voidoo^47^. To exclude the heme from the calculations, “heteroatoms” were changed to “atoms”. A computational approach using the program MOE^48^ was applied to estimate binding affinities of CYP51 enzymes for the substrates. The structures were prepared and protonated using QuickPrep (default parameters) with the heme and the substrate fixed. The energy was minimized with Amber 10:EHT force field. The sterol binding affinities were calculated as Gibbs free energies and converted into K_d_ values using the equation (ΔG =  − RT ln K_d_).

### Ferredoxin model

Because the overall fold of ferredoxins that serve as P450 redox partners is highly conserved from bacteria to mammals (e.g., 1OQQ [*Pseudomonas putida*], and 2Y5C [human], Supplementary Fig. S8), the model of *M. capsulatus* ferredoxin domain was built in Modeler (CCP4) based on the multiple sequence alignment of bacterial CYP51-fused ferredoxin domains with mammalian mitochondrial ferredoxins (Supplementary Fig. S9). The alignment has shown that the fused ferredoxins are shorter than their mitochondrial counterparts (which are all separate entities) by at least 30 amino acids at the N-terminus and 10 amino acids at the C-terminus. E.g., the lengths of human adrenodoxin is 131 residues, while the length of the *M. capsulatus* ferredoxin domain is 80 residues. Regardless of the low overall sequence identity of 12%, the three conserved cysteines of the bacterial ferredoxin domains (10, 16, and 54 in *M. capsulatus*) align with the iron-binding cysteines in the mammalian proteins (C46, C52, and C95 in human adrenodoxin), and the fourth iron-binding mammalian cysteine (C55 in human) aligns with a completely conserved bacterial glutamate (E19 in *M. capsulatus*). Although Glu as a residue coordinating an iron atom in a ferredoxin is unusual, it is found in the structures of other iron-sulfur cluster containing electron transfer proteins^49,50^. Because the bacterial fusion ferredoxins are all shorter, only the middle portion (residues 36–104) of mitochondrial ferredoxin (PDB code 2Y5C) was used for model building (Supplementary Fig. S8b).

### Docking in MOE

The P450 structure and ferredoxin model (residues 6–448 and 474–537 in the fusion protein sequence, respectively) were prepared and protonated using QuickPrep (default parameters) with the heme, lanosterol, and the [Fe2-S2] cluster tethered. The energy was minimized with Amber 10:EHT force field. The structure was then used as the receptor, with the heme bulge area (residues 385–395) being selected as the receptor site. All atoms of the ferredoxin model were used as the ligand. Patch analysis was performed using hydrophobic patch potential, the refinement was performed following the conventional rigid receptor docking protocol. 10% of the top scoring poses of the model were analyzed, and five of them that displayed the shortest distances between the P450 heme iron and the [Fe2-S2] cluster of ferredoxin are shown in Fig. 6a.

### Docking in Rosetta

Rosetta v3.11^51,52^, was used as an alternative docking approach to explore the proposed binding interface of the P450 domain more extensively. The protein interfaces for 3000 decoys were analyzed with Rosetta’s protein interface analyzer mover, then ranked by the dg_interface score term. The top 0.7% of them were filtered by the heme iron to Fe-S cluster average distance < 18 Å, five of them (interface energy range -136 to -130 Rosetta energy units) are presented in Fig. 6b.

### Electron transfer rate calculations

The rate at which electrons are transferred from a donor to an acceptor (k_et_ in s^-1^) was calculated based on the Markus theory^25^ using equation:

1 $${\text{Log}}_{{{1}0}} \,{\text{k}}_{{{\text{et}}}} = { 13 } - \, 0.{6 }\left( {{\text{R }} - { 3}.{6}} \right) \, - { 3}.{1 }\left( {\Delta {\text{G }} + \lambda } \right)^{{2}} /\lambda ,$$

where the initial constant 13 is the rate at van der Waals contact distance (R = 3.6 Å); 0.6 is the term that describes an approximately exponential fall-off in electron tunneling rate with distance through the insulating barrier; R (in Å) is the distance between the electron donor and acceptor; 3.1 is the quantized Frank–Condon factor at room temperature; ΔG is free energy (0 eV); and λ is reorganization energy (1.0 eV) [https://www.med.upenn.edu/duttonlab/golden.html].

### Molecular dynamic simulations

The two of the lowest energy decoys from Rosetta docking that best met the criteria for efficient ET were used as starting coordinates for MD simulations using AMBER 18^53^. Five N-terminal residues were deleted from the CYP51 structure, the protonation states at neutral pH were assigned their most probable values. The ff14SB force field^54^ was used for the protein. Heme parameters were taken from^55^. The iron-sulfur cluster parameters were assigned according to^56^, except the GLU-S coordination was implemented as a non-bonded interaction with a distance restraint of 2.0 Å, consistent with analogous interactions observed in the PDB. The system was solvated in an octahedral box of SPC/E water molecules that extends at least 12 Å from the protein surface and neutralized with Na^+^ counter ions. The CUDA-accelerated version of pmemd from AMBER 18^57^ was used for all calculations. Initial minimization was performed in four stages. First, solvent atoms were minimized for 20,000 steps, with protein and HET groups held close to their starting positions with a harmonic restraint of 100 kcal/mol Å2). Next, the FeS group and the chelation loop was minimized over 20,000 steps, with the solvent and remaining solute atoms treated with harmonic restraints. Then all solute atoms were minimized over 40,000 steps, with solvent treated with harmonic restraints. Finally, all atoms were minimized, unrestrained, for 50,000 steps. For each stage, 75% of the minimization cycles were performed using the steepest descents algorithm before switching to conjugate gradient for the final 25% of cycles. Careful heating to 298 K was then performed using a six-stage protocol that begins with 10,000 steps of dynamics and all atoms treated with harmonic restraints of 16 kcal/mol Å^2^. The following stages sequentially reduce the restraint force constant (4.0, 1.0, 1.0, 0.25, and finally 0.0 kcal/mol Å^2^) while increasing the number of steps of equilibration (25,000, 50,000, 100,000, 150,000, and finally 200,000). Equilibration was carried out under constant temperature, constant volume (NVT) conditions. A time step of 1.0 fs was used except in the first heating stage where we used 0.5 fs. The 600 ns production runs were performed with a time step of 1.0 fs. The non-bonded cutoff was set to 10 Å and all the bonds to hydrogen atoms were constrained with the SHAKE algorithm. Temperature was controlled at 298 K by Langevin dynamics using a collision frequency of 3.0/ps. Pressure was controlled at 1 atm using the Berendson barostat with istotropic scaling and a relaxation time of 1 ps.

## Supplementary Information

Supplementary Information.

## Data Availability

The atomic coordinates and structure factors have been deposited in the Protein Data Bank (http://wwwpdb.org) under the accession number 7SNM. The ferredoxin model and the pdb files obtained upon molecular dynamics simulations are available at request. All other data included in this article or its online Supplementary Material.
