# Supplementary material for: Unravelling the role of transient redox partner complexes in P450 electron transfer mechanics
Source: Sci Rep. 2022 Sep 28;12:16232. doi: 10.1038/s41598-022-20671-0 (PMC9519919; doi:10.1038/s41598-022-20671-0)
Supplement: Supplementary file 1 — Supplementary Information. [file 41598_2022_20671_MOESM1_ESM.pdf]

Supplementary Information:

Unravelling the role of transient redox partner complexes in P450 electron transfer mechanics

Tatiana Y. Hargrove[1], David C. Lamb[2], Jarrod A. Smith[1,4], Zdzislaw Wawrzak[3], Steven L. Kelly[2], Galina I. Lepesheva[1,4*]

[1]Department of Biochemistry, Vanderbilt University School of Medicine, Nashville, Tennessee 37232, USA

[2]Faculty of Health, Medicine and Life Science, Swansea University, Swansea, SA2 8PP, UK.

[3]Synchrotron Research Center, Life Science Collaborative Access Team, Northwestern University, Argonne, Illinois 60439, USA

[4]Center for Structural Biology, Vanderbilt University, Nashville, Tennessee 37232, USA

*Corresponding author: galina.i.lepesheva@vanderbilt.edu

This file contains:

1. Supplementary Table S1 (Data collection and refinement statistics)

2. Supplementary Fig. S1 (The asymmetric unit of *M. capsulatus* CYP51)

3. Supplementary Fig. S2 (Structure superimposition and structure-based sequence alignment)

4. Supplementary Fig. S3 (Spectral responses to the binding of sterol substrates)

5. Supplementary Table S2 (Active site cavity lining residues)

6. Supplementary Fig. S4 (Rearrangements in the *M. capsulatus* CYP51 proton relay network)

7. Supplementary Fig. S5 (A Coot snapshot of the $2F_0$-F_c density map)

8. Supplementary Fig. S6 (Molecular-dynamics trajectories)

9. Supplementary Fig. S7 (Possible ET path in *M. capsulatus* CYP51-fx)

10. Supplementary Fig. S8 (Overlaid ferredoxin structures and the *M. capsulatus* fx model)

11. Supplementary Fig. S9 (Alignment of bacterial CYP51-fused and mammalian ferredoxins)

Supplementary Table S1. Data collection and refinement statistics.

PDB ID	7SNM
Data collection	
Wavelength, Å	1.000
Space group	P_12_11
Cell dimensions	
a, b, c, Å	73.2 206.20 81.4
α, β, γ, °	90.0 114.1 90.0
Molecules per asymmetric unit	4
Resolution (upper shell), Å	74.45-2.55 (2.62-2.55)
Solvent content, %	47.7
R_{merge} (upper shell)	0.065 (0.637)
CC (1/2) (upper shell)	0.998 (0.960)
I/σ(I) (upper shell)	13.4 (2.2)
Completeness (upper shell), %	99.0 (99.9)
Redundancy (upper shell)	7.1 (7.4)
Refinement	
No. of reflections	67008
R_{work}/R_{free}	0.204/0.246
R.m.s deviations	
Bond lengths, Å	0.002
Bond angles, °	1.3
Ramachandran plot	
Favorable/allowed, %	94.0/99.8
Outliers, %	0.2
Average B factor, Å^2	54.9
Model	
No. of atoms	14767
No. of residues per molecule	
Protein (B factor, Å^2, A/B/C/D)	443 (45.5/48.6/66.6/77.7)
Heme (B factor, Å^2)	1 (13.8/16.2/35.5/31.0)
Water (B factor, Å^2)	341 (47.1)
Lanosterol [Lan] (B factor, Å^2)	1 (13.5/20.0/31.0/34.3)

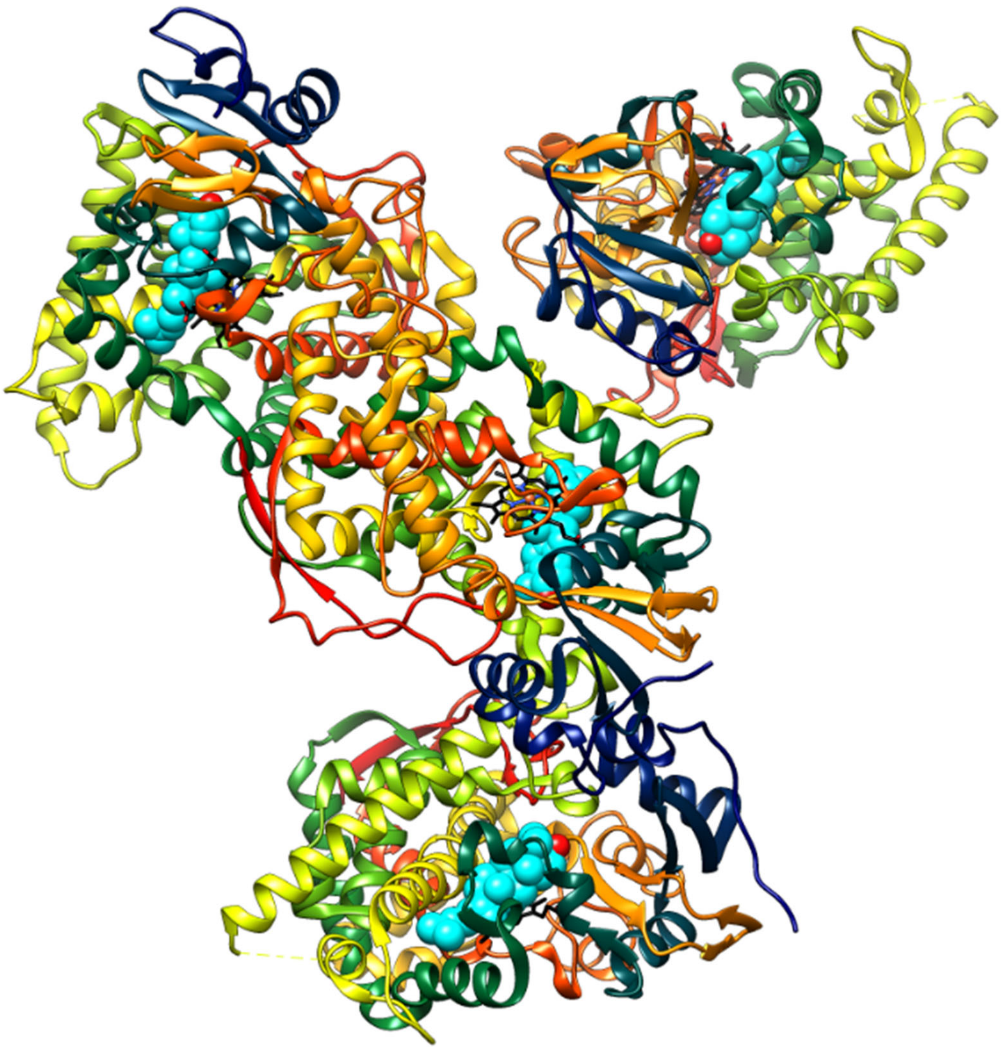

Supplementary Figure S1. The asymmetric unit of *M. capsulatus* CYP51. Four polypeptide chains of the P450 molecule are shown in a rainbow-colored ribbon representation, from the N-terminus (blue) to the C-terminus (red). The heme is seen in a stick representation, the carbon atoms are black. The atoms of lanosterol are presented as spheres, the carbons are cyan. The RMSD of Cα between the four molecules is 0.35 Å.

a

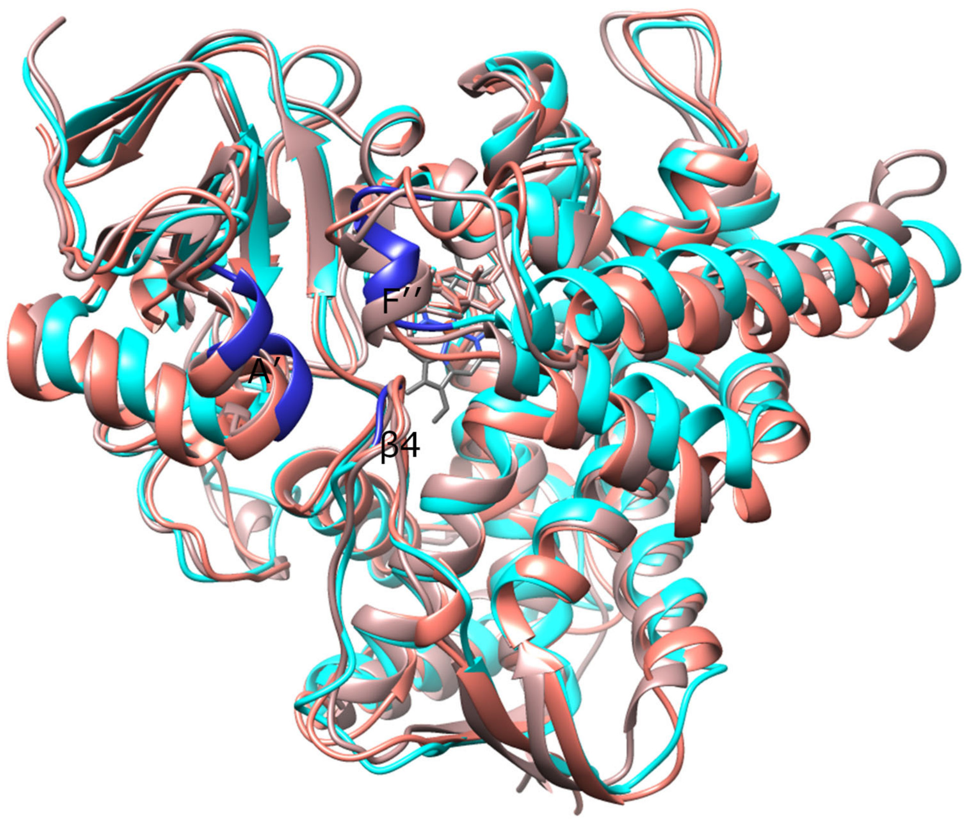

b

```
Conservation:                  4994 4   949994  99949 494         4 94 4 4 444 4 4 44 4   44    49 9     49 4 499499 9944444 44  494 49   9 44 4   4
6uez_chainA_p002      1   KGKLPPYIFSPIPFLGHAIAFGKSPIEFLENAYEKY-GPVFSFTMVGKTFTYLLGSDAAALLFNSKNEDLNAEDVYSRLTTPVFGKGVAYDVPNPVFLEQKKMLKSGLNIAHFKQHV   116
6fmo_chainA_p003      1   ---LPPVYPVTVPFLGHIVQFGKNPLEFMQRCKRDLKSGVFTISIGGQRVTIVGDPHEHSRFFSPRNEILSPREVY-TFMTPVFGEGVAYAAPYPRMREQLNFLAEELTIAKFQNFV   113
7snmA.pdb_chainA_s001 1   -SNTPPVKPGGLPLLGHILEFGKNPHAFLMALRHEF-GDVAEFRMFHQRMVLLTGSQASEAFYRAPDEVLDQGPAY-RIMTPIFGRGVVFDARIERKNQQLQMLMPALRDKPMRTYS   114
Consensus_aa:             ...LPPVhPts lPFLGHIlpFGKNPlEFLpst++-@.GsVFoFoMhGQRhTlLhGScAtthFFst+NElLssc-VY.RlMTPVFG+GVAYDAPhPRhpEQLpMLhstLsIA+F+s@V
Consensus_ss:                                 hhh    hhhhhhhhhhhh     eeeeee  eeeeee  hhhhhhhhh   hhh       hhhhh    eee    hhhhhhhhhhhhh  hhhhhhhh

Conservation:              49  94         4 4 9 44449    4449 944499 9 4 9  99          4   4 4    4  449    9    44         4 4 9 4   44 4 4 4         4
6uez_chainA_p002      117 SIIEKETKEYFES--WGESGEKNVFEALSELIILTASHCLHGKEIRSQLN----EKVAQLYADLAGGFSHAAWLLPG-WLP----LPSFRRRDRAHREIKDIFYKAIQKRRQS---Q   219
6fmo_chainA_p003      114 PAIQHEVRKFMAENWKEDEGVINLLEDCGAMIINTACQCLFGEDLRKRLNARHFAQLLSKMES---SLIPAAVFMPWLLRLP--LPQSARCREARAELQKILGEIIVAREKEEASKD   225
7snmA.pdb_chainA_s001 115 EIIVAEVEAMLRD--WKDAGTIDLLELTKELTIYTSSHCLLGAEFRHELN----TEFAGIYRDLEMGIQPIAYVFP--NLPLPVFKRRDQARVRLQELVTQIMERRARSQ--E----   217
Consensus_aa:             sIIp+EV+c@hc-..WcDtGhINLLEhhtELIIhTASHCL@GcElR+cLN....hclAtlYcD...GlpPAA@lhP..hLP....bpShRtR-RhhELlKpIhE+hlp+c..E....
Consensus_ss:             hhhhhhhhhhhhh  hh     hhhhhhhhhhhhhhhhh          h     hhhhhhhhhhhhhhhhhhhhhhh  hh      hhhhhhhhhhhhhhhhhhhhhhhhhhhh

Conservation:                   4 44 9449 9 99  44 494 99444  49944994 9449     9 4   4  4 4    944          4444 4   44 4444449 494 994   9944
6uez_chainA_p002      220 EKIDDILQTLLDATYKDGRPLTDDEVAGMLIGLLLAGQATSSTTSAWMGFFLAR--DKTLQKKCYLEQKTVCGENLPPLTYDQLK-DLNLLDRCIKETLRLRPPIMIMMRMARTPQT   333
6fmo_chainA_p003      226 NNTSDLLGGLLKAVYRDGTRMSLHEVCGMIVAAMFAGQHTSTITTSWSMLHLMHPKNKKWLDKLHKEIDEF----PAQLNYDNVMDEMPFAERCVRESIRRDPPLLMVMRMVKAEVK   338
7snmA.pdb_chainA_s001 218 -RSTNVFQMLIDASYDDGSKLTPHEITGMLIATIFAGHHTSSGTTAWVLIELLR--RPEYLRRVRAEIDALFET-HGRVTFESLR-QMPQLENVIKEVLRLHPPLILLMRKVMKDFE   329
Consensus_aa:             .+ooDlLQhLLDAoY+DGo+LTsHEVtGMLIAhlFAGQHTSShTTAWhhlcLhR..cKc@L+Kl+hEIDhl....htpLTYDsL+.-MPhLERCIKEoLRL+PPLlllMRMV+h-hc
Consensus_ss:                  hhhhhhhhhh        hhhhhhhhhhhhh    hhhhhhhhhhhhhhhh   hhhhhhhhhhhhhh         hhhhh   hhhhhhhhhhhhh  hhhh         ee

Conservation:             9 44 44 9   44 49 4 44   4 4444 4449494        4    4    9949494949  99   94949  4 49 949 9       4  49 4944 9   4 4449  4
6uez_chainA_p002      334 VAGYTIPPGHQVCVSPTVNQRLKDSWVERLDFNPDRYLQDNPASGEKFAYVPFGAGRHRCIGENFAYVQIKTIWSTMLRLYEFDLIDGY-FPTVNYTTMIHTPEN-P-VIRYKRRS-   446
6fmo_chainA_p003      339 VGSYVVPKGDIIACSPLLSHHDEEAFPNPRLWDPERD------EKVDGAFIGFGAGVHKCIGQKFALLQVKTILATAFREYDFQLLRDE-VPDPDYHTMVVGPTLNQCLVKYTRKKK   448
7snmA.pdb_chainA_s001 330 VQGMRIEAGKFVCAAPSVTHRIPELFPNPELFDPDRYTPERAEDKDLYGWQAFGGGRHKCSGNAFAMFQIKAIVCVLLRNYEFELAAAPESYRDDYRKMVVEPAS-PCLIRYR-RR-   443
Consensus_aa:             VtGYhIPsG+hVCtSPoVoHRlcEtFPNPcLFDPDRY......-K-c@A@ltFGAGRHKCIGpsFAhlQIKTIltThLRpYEF-Llctb.hPcsDY+TMVVsPhs.P.LIRY+.R+.
Consensus_ss:             e  eee    eeee hhhh    hh                     ee            hhhhhhhhhhhhhhhhhheeeee                eeee      eeeeeee
```

Supplementary Figure S2. a. Superimposition of the substrate-bound *M. capsulatus*, human, and *T. cruzi* CYP51s. The coloring is as in Fig. 3b. The secondary structural elements that form the substrate entry (helices A′, F′′, and the β4 hairpin) are colored in blue on the *M. capsulatus* structure. **b**. Structure-based sequence alignment (PROMALS3D). Sequences are colored according to secondary structures (red: alpha-helix, blue: beta-strand). Conservation indices above 3 are shown.

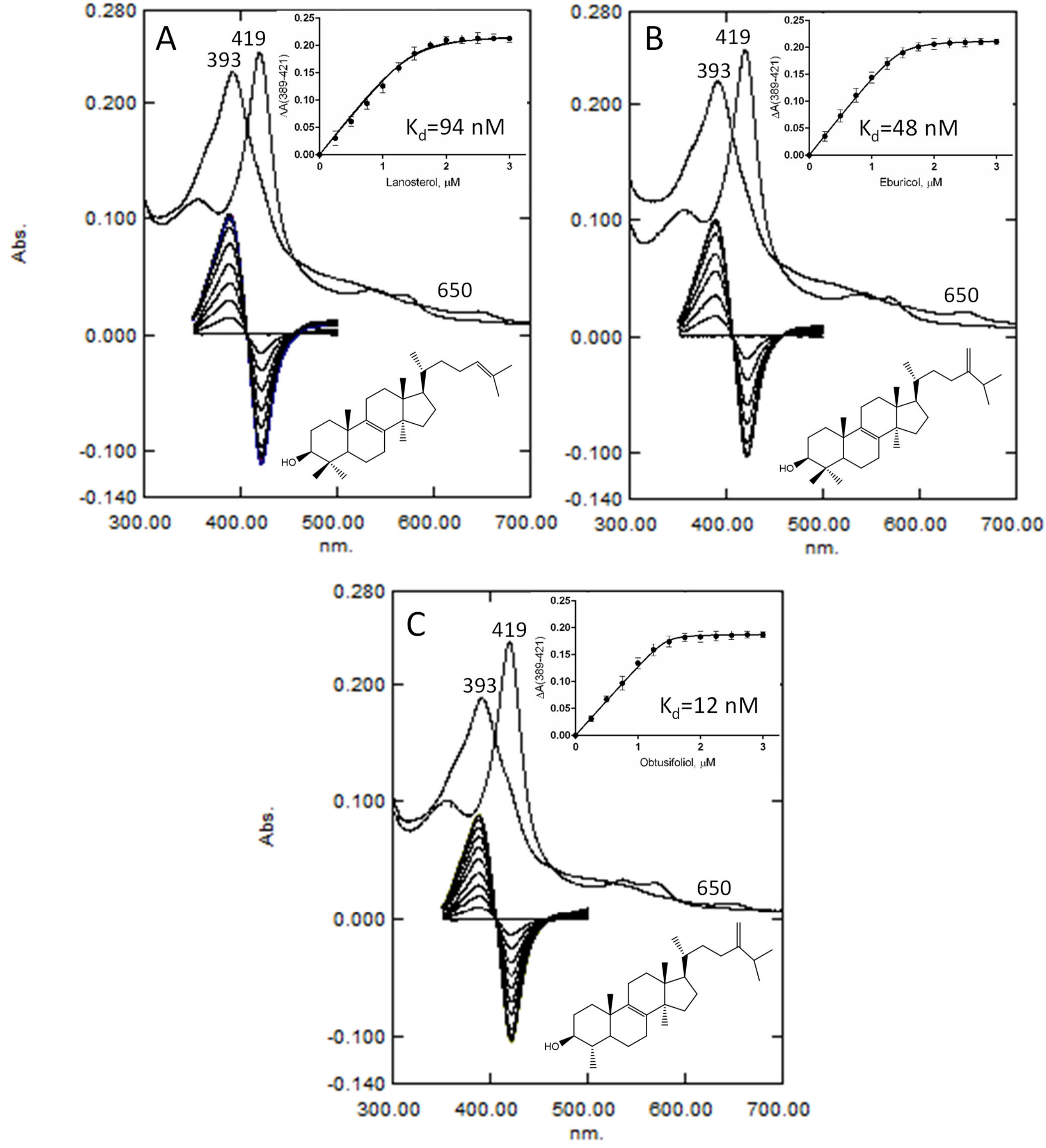

Supplementary Figure S3. **Spectral changes observed during titration of *M. capsulatus* CYP51-fx with (A) lanosterol (B) eburicol, and (C) obtusifoliol**. Absolute (top) and difference (bottom) spectra. The P450 concentration was ~2 μM. Inset: The titration curves fitted to the quadratic (Morrison) equation.

Supplementary Table S2. Active site cavity lining residues in the substrate-bound bacterial (*M. capsulatus*, 7SNM), protozoan (*T.cruzi*, 6FM0), and mammalian (human, 6UEZ) CYP51 structures

Structural element	*M. capsulatus*	Human<br>D231A/H314A	*T. cruzi*<br>I105F
	Substrate<br>(Lanosterol)	Substrate<br>(Lanosterol)	Substrate<br>(Obtusifoliol)
B' helix	**Y79**<br>I81<br>M82<br>**F86**	**Y131**<br>L134<br>T135<br>**F139**	**Y103***<br>F105<br>M106<br>**F110**
B'' η-turn	**V90**<br>V91<br>F92	**V143**<br>A144<br>Y145	**V114**<br>A115<br>Y116
C helix	K99<br>**Q102**<br>L103<br>M105<br>**L106**<br>M107	F152<br>**Q155**<br>K156<br>M158<br>**L159**	M123<br>**Q126**<br>L127<br>F129<br>**L130**
F'' helix	I181<br>Y186	F234	M204<br>S207<br>L208<br>I209<br>P210<br>V213
G' helix			L219
I helix	**G249**<br>**M250**<br>L251<br>A253<br>T254<br>F256<br>**A257**<br>**H260**<br>**T261**	**G303**<br>**M304**<br>G307<br>L308<br>L310<br>**A311**<br>**A314**(H314)**<br>**T315**	**G283**<br>**M284**<br>I285<br>A287<br>A288<br>F290<br>**A291**<br>**H294**<br>**T295**
K/b1-4 loop	**P321**<br>L322<br>I323<br>*L324****	**P376**<br>I377<br>M378<br>*L379****	**P355**<br>L356<br>L357<br>*M358****
β1-4	L325<br>**M326**	M380<br>**M381**	V359<br>**M360**
β4 hairpin	**M433**<br>V434	**M487**<br>I488	**M460**<br>V461
Total #,<br>volume, Å^3	32<br>913	29<br>865	36<br>1203

*Residues invariant across the biological kingdoms (**in bold**), **mutant (H314A), ***H-bond with the substrate OH group (*italic*)

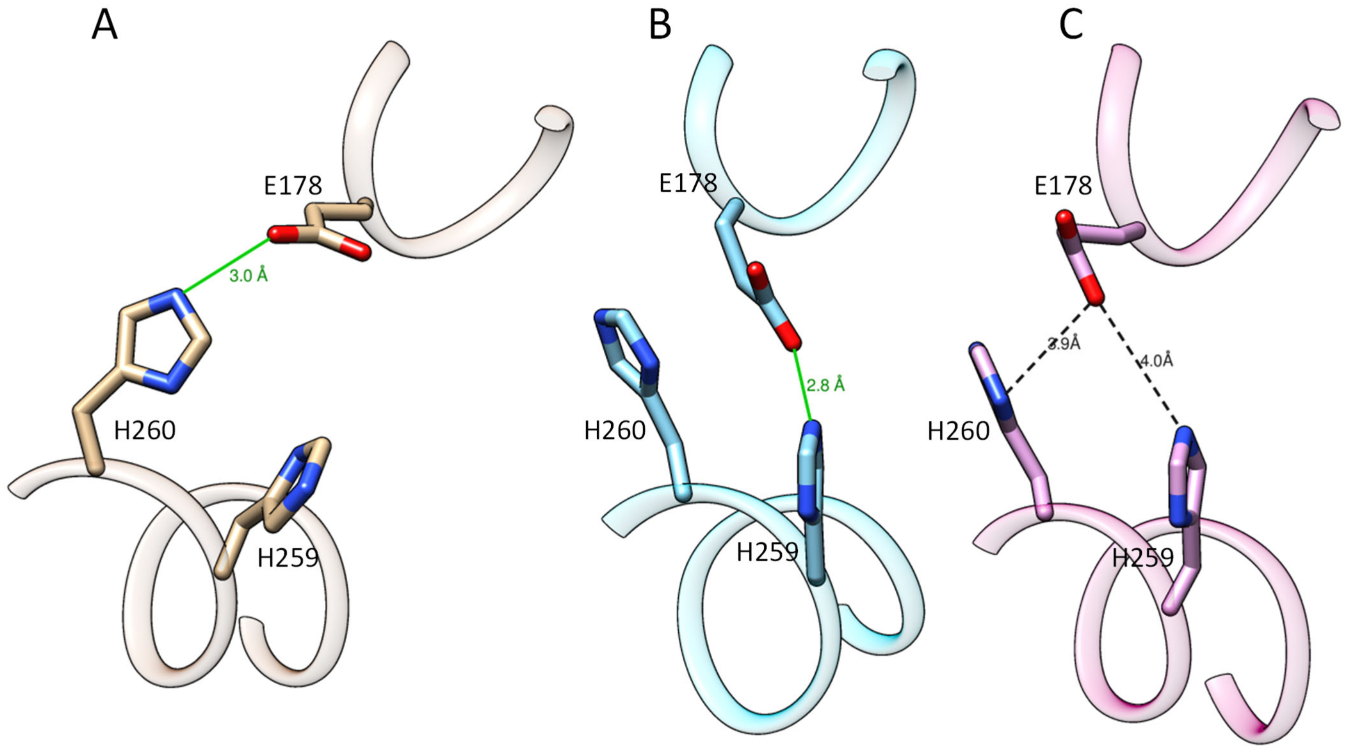

Supplementary Figure S4. **Rearrangements in the proton relay network in *M. capsulatus* CYP51.** Ligand-free (salt bridge E178-H260, 3.0 Å) (**A**), lanosterol-bound (salt bridge E178-H259, 2.84 Å) (**B**) structures of *M. capsulatus* CYP51, and a snapshot from MD simulations of the CYP51-ferredoxin complex, |Fe-Fe1|=16.6 Å. (E178 is freed, proton delivery route activated) (**C**).

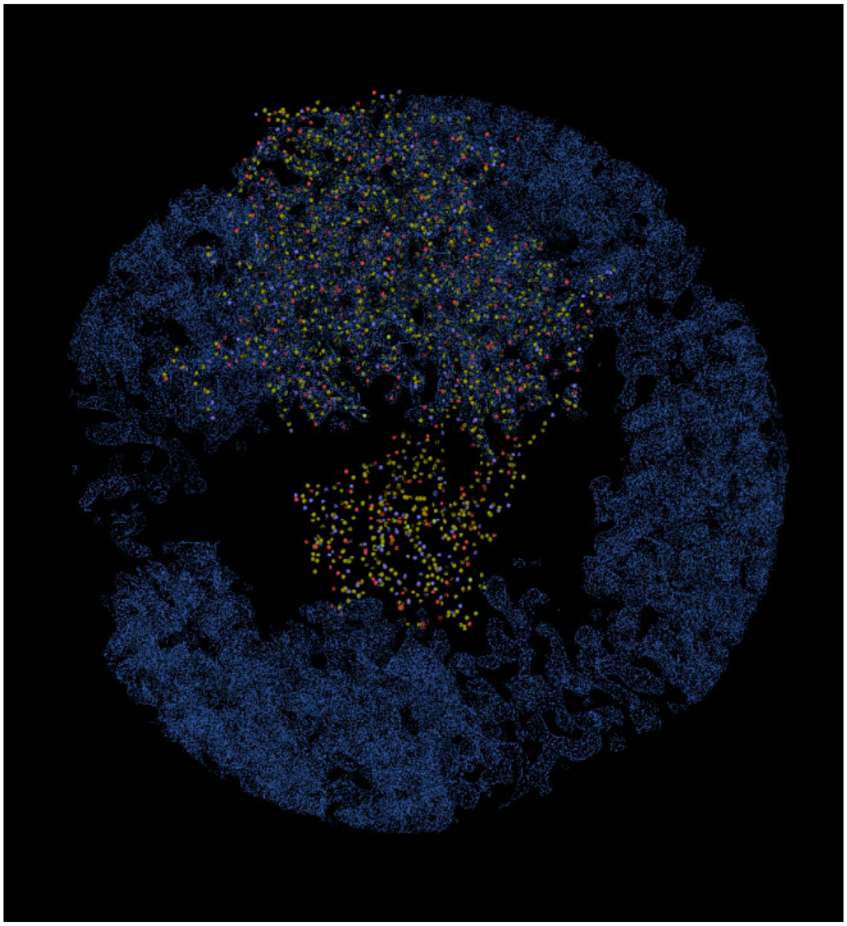

Supplementary Figure S5. **A Coot snapshot of the $2F_0$-F_c density map contoured at 1.5 σ, map radius 50 Å.** The dots correspond to one molecule of P450 (top, proximal face down) and one molecule of ferredoxin (bottom, the docked pose, selected for MD simulations). The electron density is clearly seen for the P450 domain only, while any density close to the proximal P450 surface (where the ferredoxin domain is supposed to interact) is absent.

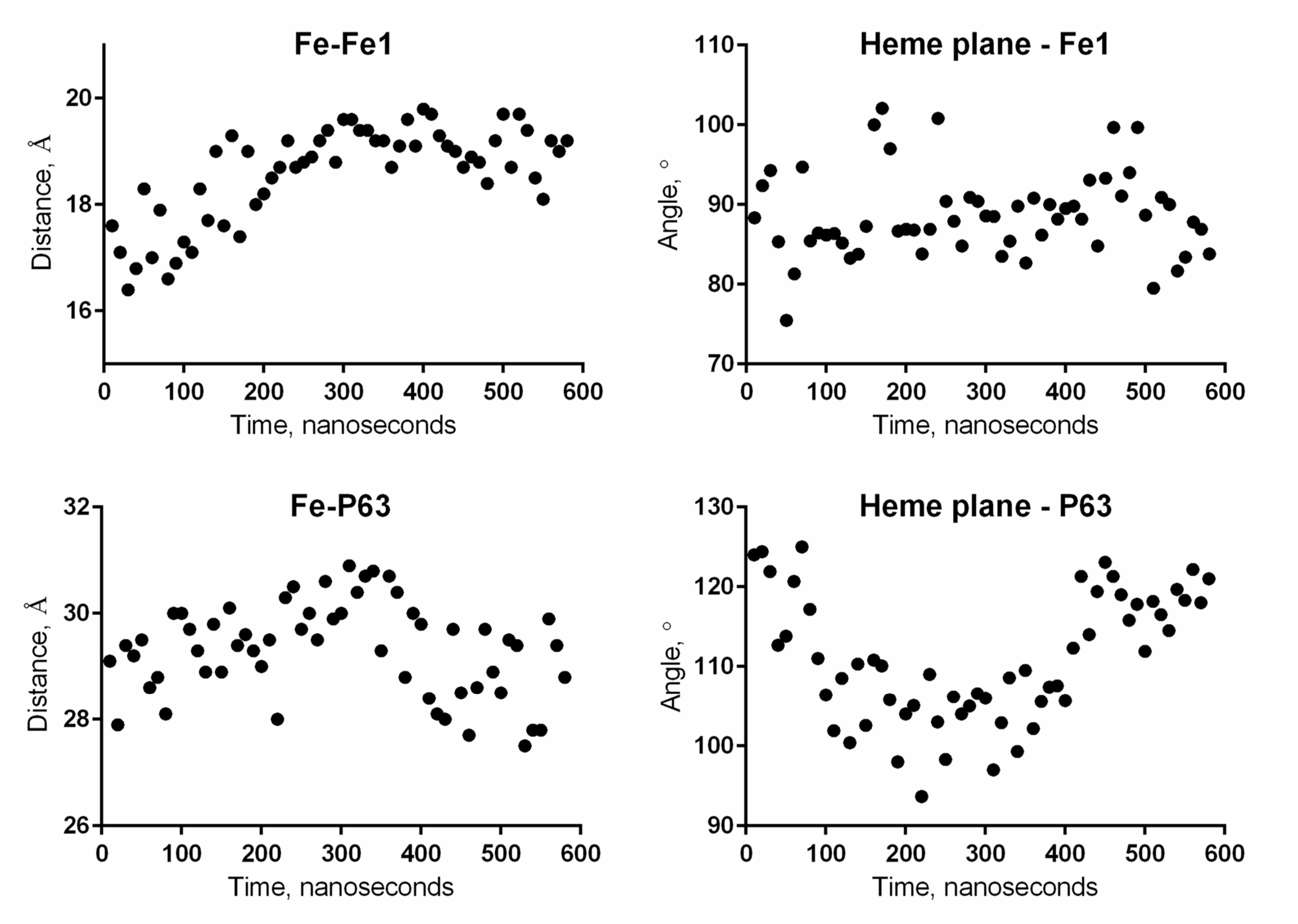

Supplementary Figure S6. Molecular-dynamics trajectories. The pbd files were written each 10 ns. The maximal Fe-Fe1 distance of 19.8 Å (at 400 ns of the 600 ns run) is perfectly compatible with electron transfer, the calculated K_{ET} value being 1.51/sec (or 90.8 electron transfer events per minute).

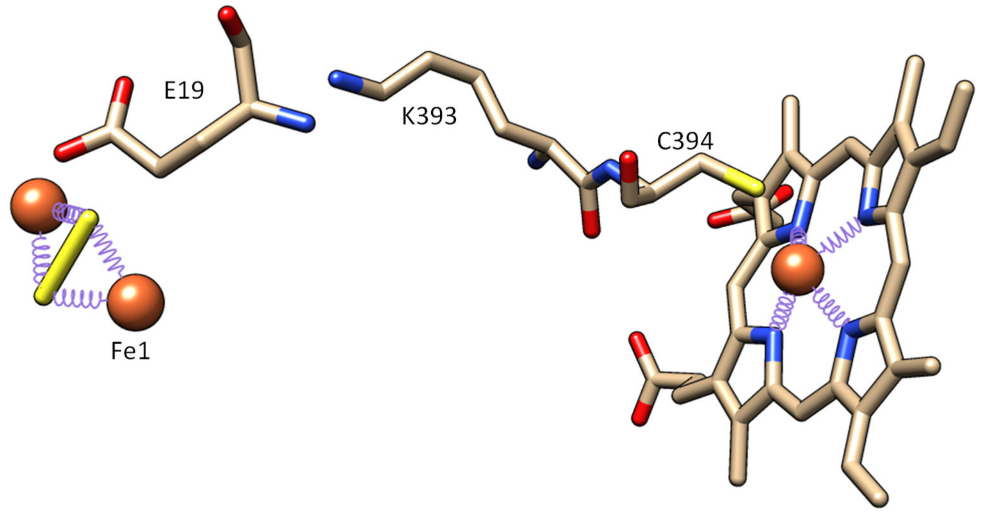

Supplementary Figure S7. Possible ET path in *M. capsulatus* CYP51-fx.

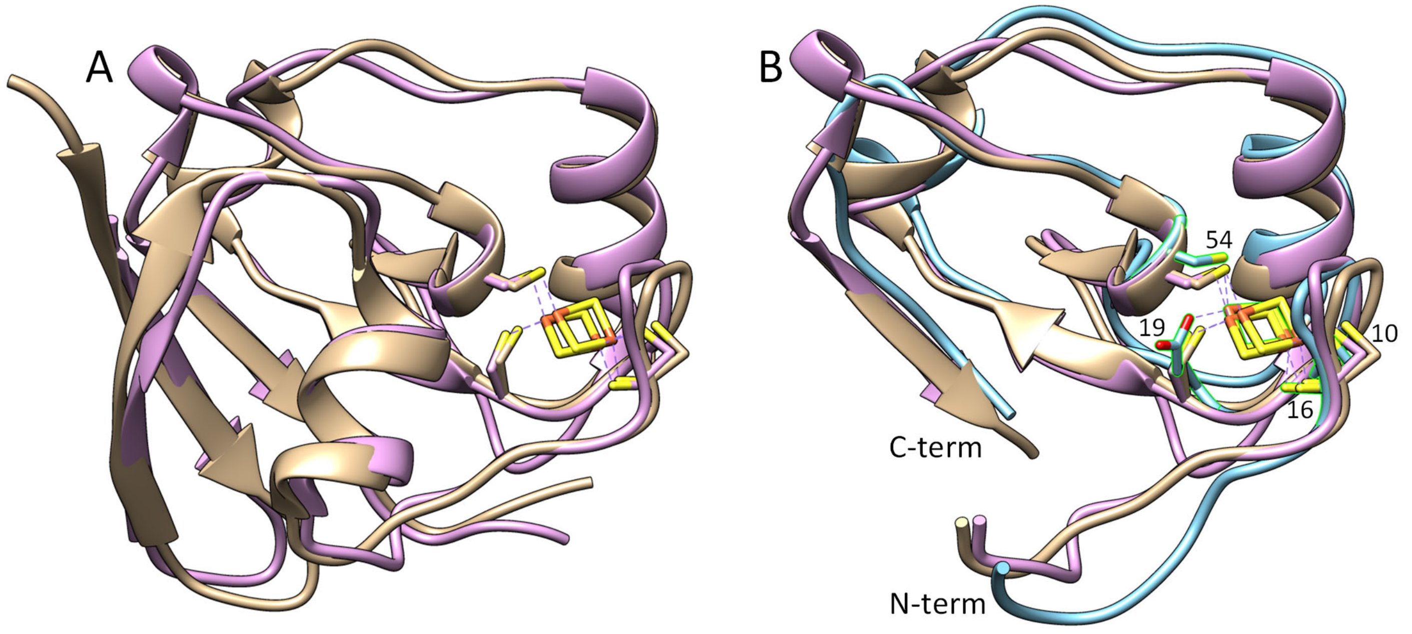

Supplementary Figure S8. **Ferredoxins that serve as P450 redox partners display highly conserved structural fold**. **A**. Overlaid structures of putidaredoxin from *Pseudomonas putida* [1OQQ, tan] and adrenodoxin from human [2Y5C, plum]. **B**. The 35-103 fragments of the same structures overlaid with the model of *M. capsulatus* ferredoxin fusion (blue). The 2Fe-2S cluster, Cys10, 16, 54, and Glu19 of the model are selected (in green) and the residues are numbered.

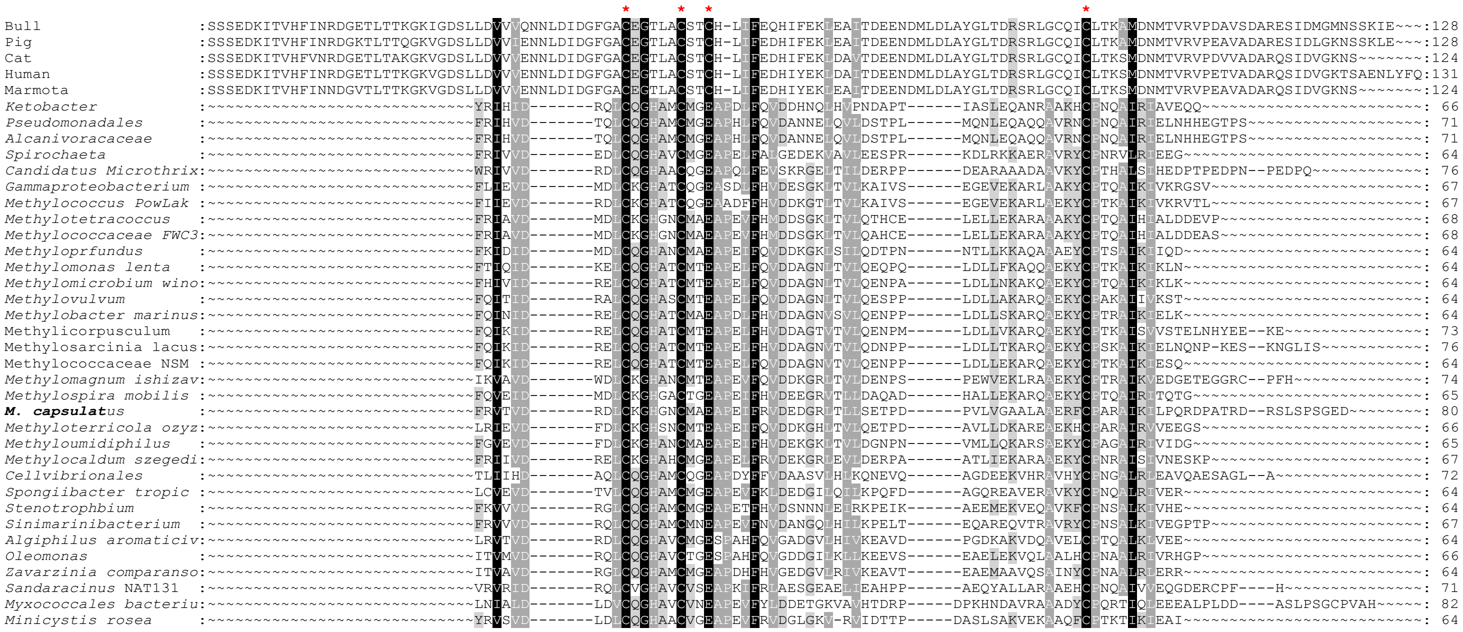

Supplementary Figure S9. Alignment of 5 mammalian ferredoxins and 30 bacterial ferredoxin domains that exist as naturally fused C-terminal portion of CYP51 genes. Four residues that form the interactions with the [Fe_2-S_2] cluster are marked with red stars.
